# The effect of Bach flower remedies on hope and quality of life in people with advanced cancer: a triple-blind randomized clinical trial

**DOI:** 10.1590/1980-220X-REEUSP-2025-0451en

**Published:** 2026-07-20

**Authors:** Leonel dos Santos Silva, Amanda Gomes de Miranda, Larissa Marcondes, Ruth Natalia Teresa Turrini, Paulo Ricardo Bittencourt Guimarães, Maria de Fátima Mantovani, Luciana de Alcantara Nogueira, Luciana Puchalski Kalinke

**Affiliations:** 1 Universidade Federal do Paraná, Programa de Pós-Graduação em Enfermagem, Curitiba, PR, Brazil.; 2 Universidade de São Paulo, Escola de Enfermagem, Departamento de Enfermagem Médico-Cirúrgica, São Paulo, SP, Brazil.; 3 Universidade Federal do Paraná, Departamento de Estatística, Curitiba, PR, Brazil.

**Keywords:** Flower Essences, Hope, Quality of Life, Integrative Oncology, Palliative Care

## Abstract

**Objective:**

To evaluate the effect of Bach Flower Remedies Therapy on the hope and quality of life of people with advanced cancer.

**Method:**

Randomized, triple-blind (1:1) clinical trial in a high-complexity public hospital in the Southern region of Brazil. Ninety-nine participants were allocated to either the intervention group (solution with nine flower essences) or the placebo group (Hydro-brandy solution). Both groups used four drops orally, four times a day, for 120 days, with monthly follow-up. Hope measured by the *Herth Hope Index,* quality of life through *Functional Assessment of Chronic Illness Therapy* – *Palliative Care*.

**Results:**

In the flower remedies group (n = 50), hope varied from 43.44/48 to 44.53/48 and in the placebo group (n = 49) from 41.67/48 to 44.29/48, a significant increase over time (p = 0.024), with no difference between groups (p = 0.977). Quality of life ranged from 141.39/184 to 150.60/184 in the flower remedies group, and from 133.76/184 to 148.96/184 in the placebo group, with no significant difference between groups (p = 0.230) or time (p = 0.240).

**Conclusion:**

Although not statistically significant, there was a slight increase in life expectancy and quality of life, highlighting the importance of non-pharmacological therapies in evidence-based palliative care practice.

**Brazilian Registry of Clinical Trials:**

RBR-8q6z6kq.

## INTRODUCTION

Between the narrow dichotomous view of curable and incurable, advanced disease is intrinsically associated with suffering and coping. In advanced stages of cancer, the Palliative Treatment (PT), Palliative (PC) and Supportive Care aim to improve Health-Related Quality of Life (HRQoL) and extend survival^(1,2)^.

Regardless of the stage of the disease or therapeutic objectives, early PC is indicated because it adopts a person- and family-centered approach, with a transformative perspective that moves beyond a disease-centered model, addressing the biopsychosocial-spiritual dimensions to maintain survival with quality^(1,2,3)^. Many people are on PT without early integration with PC approaches, even though they share goals to benefit patients in eligible clinical conditions.

In advanced or incurable cancer, different types of PT are indicated, whether systemic (chemotherapy, hormone therapy, immunotherapy, and targeted therapy) or localized (radiotherapy and surgery), aimed at controlling the progression of metastases. These are complex therapies, managed in outpatient or inpatient units by transdisciplinary teams, which should be offered in a timely manner and concurrently with a PC approach^(2)^.

In the context of palliative issues, scientific production is largely directed towards investigating and optimizing HRQoL, understood as the person’s perception of the impact of illness and treatment on the physical, functional, social, psycho-emotional, existential and of finitude multidimensionality^(1,3,4)^. However, in people with advanced cancer, hope remains under-researched, being highlighted in only 4% of publications^(3)^. Hope coexists with health-illness-chronic care; it is a coping mechanism that has to be evaluated and promoted^(5)^.

Hope is the driving force for survival, not limited by traits, linear concepts, or externally determined, understood as “a vital and dynamic force […] characterized by confident expectation…” ^(6)^it is “a motivational and cognitive attribute […] to initiate and maintain action towards achieving the goal”^(7)^. Hope is related to sociodemographic, clinical, and therapeutic factors, HRQoL, existential issues, social support, positive psychology, resilience, coping, among others^(5,8,9)^.

Psycho-Oncology and Integrative Oncology are continuously dedicated to the search for non-invasive and non-pharmacological interventions for human health with scientific basis, safety and efficacy, here called Non-Pharmacological Therapies (NPT), to provide holistic care^(4)^. Among the 604 different classified NPTs, the Essences or Bach Flower Remedies (BFR) were developed in England between 1928 and 1935 by the homeopathic physician Edward Bach^(10)^. These are solutions highly diluted in water, extracted from wild plants and flowers, intended for psycho-emotional balance, acting on subtle levels of internal harmonization of the person and environment^(11,12)^.

Whether pharmacological traces remain is unclear, thus there is no consensus on the BFR’s mechanism of action. One hypothesis is that the ultra-diluted solution contains nanoparticles with subtle information/energy from the flowers and has a psychomodulatory action on the conscious and unconscious mind, to rebalance emotional states^(11,13,14,15)^.

Even in the face of uncertainties related to the mechanism of action of BFR, it is recognized by the World Health Organization (1976), authorized by the National Policy on Integrative and Complementary Practices (2018), and supported by the Federal Nursing Council. It is used in society with the expectation of some result; consequently, some studies underscore its effects on attention deficit hyperactivity disorder^(13)^; for conditions that interfere with the body and mind^(12)^; in reducing stress, anxiety, compulsive eating, and heart rate^(16)^; and in mental health, insomnia, and emotion management^(15)^.

Despite the growing interest in integrative Oncology, there are few randomized clinical trials evaluating the effect of Bach Flower Remedies on advanced cancer, focusing on hope and quality of life. In this context, this study aimed to evaluate the effect of Bach Flower Remedies Therapy on the hope and quality of life of people with advanced cancer.

## METHOD

### Design of Study

Randomized, triple-blind, placebo-controlled clinical trial conducted in the Oncology-Hematology outpatient clinic of a public hospital in the Southern region of Brazil. Two guidelines were followed when reporting this essay: *Consolidated Standards of Reporting Trials* (CONSORT) and its extension to *Nonpharmacologic Treatments* (NPT)^(17)^.

### Participants and Sample

The volunteer participants included adults (≥ 18 years) with advanced cancer (stage IV), classified on the scale *performance status of the Eastern Cooperative Oncology Group* (PS-ECOG) from 0-3. Cases of lymphoproliferative diseases, patients exclusively on Palliative Care, and those without verbal and/or written communication abilities were excluded.

The sample size was calculated in the software GPower (v 3.1.9.7) for longitudinal model (2 groups and 5 measurements), assuming f = 0.25, α = 5% and power of 95%. Taking into account anticipated losses, the plan was to recruit 125 patients. Recruitment took place from October 2022 to February 2024, with follow-up evaluations until June 2024.

### Ethical Aspects

The study was approved by the institutional research ethics committee with opinion number 5.204.355 and registered in the Brazilian Registry of Randomized Clinical Trials (ReBEC) under opinion number RBR-8q6z6kq. All participants received verbal and written explanations and signed an informed consent form before being included in the study.

### Intervention

All vials were identical, made of sterile amber glass, with a capacity of 30 mL, a perforated cap, a safety seal, a dropper with a white bulb, and a 75 mm glass cannula. The prepared solutions could contain either the flower formula (FG) or the placebo solution (PG). They were all similarly labeled “Flower Research” and included instructions for use, differing only in the numerical and sequential labeling.

The solutions were prepared in a specialized pharmacy, in identical 30ml vials. Each vial contained enough product for 30 days of use, with an additional 30% margin. The flower group received hydro-brandy with nine Bach flower essences, while the placebo group received only hydro-brandy. Both groups took four drops, four times a day, for 120 days. Messages were sent via WhatsApp® during the first eight weeks to encourage participation.

The hydro-brandy solution consisted of 21 mL of mineral water (Crystal® brand, density 1.0 and pH at 25ºC = 8.71) + 9 mL of preservative brandy (Osborne® brand, 100% wine distillate and 36% alcohol by volume). The flower solution consisted of 2 drops of the mother flower essence (with 400 parts organic brandy in one part of Healing Herbs® mother essence from England). The formula (and the main indications) consisted of ***Cherry Plum*** (*Prunus cerasifera,* emotional self-control), ***Crab Apple*** (*Malus pumila,* acceptance and self-esteem), ***Gorse*** (*Ulex europaeus,* faith and hope), ***Mimulus*** (*Mimulus guttatus*, acceptance), ***Mustard*** (*Sinapis arvensis*, melancholy), ***Olive*** (*Olea europaea,* physical exhaustion), ***Sweet Chestnut*** (*Castanea sativa*, coping with adversity), ***White Chestnut*** (*Aesculus hippocastanum*, discernment) and ***Wild Oat*** (*Bromus ramosus,* purpose and meaning)^(11)^.

Both groups continued to receive palliative cancer treatment, whether systemic or localized, depending on each individual’s clinical condition.

### Outcomes

The primary outcome was assessed using the Herth Hope Index (HHI), the most widely summarized instrument in systematic reviews on this construct in oncology^(8,9)^. This is a brief assessment of self-reported hope, consisting of 12 items on a Likert scale. The total score ranges from 12 to 48, being the sum of the possible ranges, with higher scores indicating greater hope^(7)^. It can be classified into three levels of hope based on its scores: low (12–23), medium (24–35), or high (36–48). The internal consistency of the instrument, estimated by Cronbach’s α in the original version, was 0.97 (excellent)^(7)^, 0.834 (good) in its validation and cultural adaptation in Brazil^(18)^.

The secondary outcome assessed HRQoL using the self-report instrument *Functional Assessment of Chronic Illness Therapy - Palliative Care* (FACIT-Pal, version 4). FACIT-Pal is composed of the four dimensions of well-being of *Functional Assessment of Cancer Therapy* (FACT-G): physical (PWB: 7 items, 0-28 points); social/family (SWB: 7 items, 0-28 points), emotional (EWB: 6 items, 0-24 points), functional (FWB 7 items, 0-28 points) and a palliative care domain subscale (PalS 19 items, 0-76 points)^(19)^. Items are scored using a Likert scale from 0 (not at all) to 4 (very much) points; higher scores reflect better HRQoL. The subscales result in the Trial Outcome Index (TOI): PWB + FWB + PalS = 0 to 132 points) and in the total score (0-184 points)^(19)^. The reliability of the internal consistency of Cronbach’s α ranges from good to excellent in the original version of the subscales (FACT-G: 0.89; TOI: 0.91 and FACIT-Pal: 0.93)^(19)^ and in its translation into Portuguese (FACT-G: 0.91)^(20)^.

Adverse events were monitored at all visits, classified by severity and causal relationship. Serious events led to discontinuation of the intervention, while maintaining an intention-to-treat analysis.

### Data Collection

Data collection was performed using REDCap®. During participant enrollment, the following instruments were administered in person for obtaining: sociodemographic and clinical data; hope level; and HRQoL (baseline, T_0_). In the follow-up assessments, every approximately 30 days, the hope and HRQoL instruments were applied (T_1_ T_4_ – 120 days), whether in person, by phone, or by sending a link to the participant.

Participants who were absent approximately 30 days after T_0_ or the latest follow-up assessments (T_1_ to T_4_), who did not consume more than 70% of the vial’s content as reported verbally, or in case of any related adverse event, were discontinued.

### Randomization and Allocation

A random allocation sequence with 150 numbers (1 for intervention and 2 for placebo) was website-generated (https://ctrandomization.cancer.gov/tool/), preserving the 1:1 allocation balance between the groups, a procedure performed by a non-blinded researcher (without contact with the participants or field researcher). Randomization was provided to the pharmacist (without contact with participants or field researcher) in charge of handling the vials. These numerical codes were used to identify the vials, determine the contents (BFR or placebo), and ensure allocation blinding (for both participants and field researcher).

### Blinding

Triple blinding (participants, field researcher, and statistician) was maintained until the end of the study. A non-blinded researcher performed the randomization, and the pharmacist handled the manipulation; neither of them had contact with the participants, the field researcher, nor the statistician. The compounding pharmacy was located far from the data collection clinic. The field researcher maintained contact with the dispensing team and was responsible for data collection. After data collection was complete, the field researcher forwarded the data to the randomization researcher, who, after randomization, organized the data into groups A and B and sent them to the statistician. After the analyses, the non-blinded researcher clarified which group received the flower essence and which received the placebo.

### Data Analysis

The captured data were exported from REDCap® and analyzed using the software SPSS® 20. To verify the homogeneity of the groups, the Chi-square or Fisher's exact test was used for qualitative variables, and the Student's t-test was used for quantitative variables.

The analyses followed the intention-to-treat principle to minimize selection bias after randomization, associated with the Generalized Linear Mixed Model (GLMM), due to the need to incorporate all available assessments of each participant over time up to the moment of discontinuation, according to the maximum likelihood principle^(21)^. An unstructured covariance matrix and the Akaike Information Criterion (AIC) were adopted for model selection. The significance level was set at 5%, with Bonferroni correction for multiple comparisons.

The correlations between hope scores and FACIT-Pal were verified using Spearman’s coefficient. The significance level was set at 5%.

## RESULT

**Figure 1:**
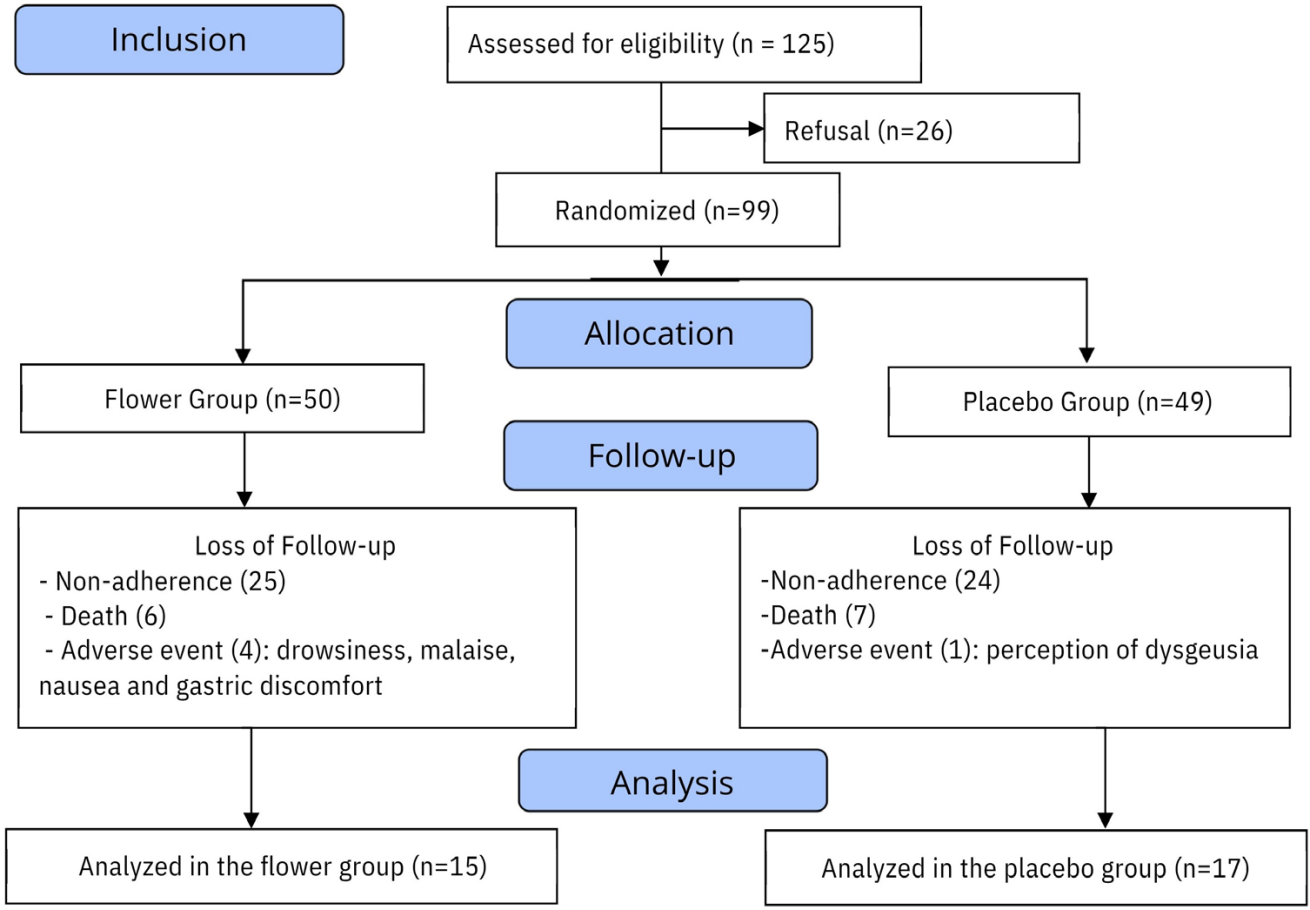
Flowchart of participants.

Of the 125 eligible patients, 99 were randomized (FG = 50, PG = 49). Losses were higher in the initial phases, mainly due to non-participation or death. At the end of the segment, 15 participants were analyzed in the FG and 17 in the PG (Figure 1). The deaths were due to the progression of the disease or complications related to treatment, unrelated to the intervention itself.

### Participant Characteristics

The groups were homogeneous with respect to sociodemographic, clinical, and therapeutic variables (p > 0.05) (Table 1).

**Table 1:** Characteristics of participants in the Flower Group (FG) and Placebo Group (PG) – Curitiba, PR, Brazil, 2025.

	Flower group (n = 50)		Placebo group (n = 49)		*p*
	n	(%)	n	(%)	
Women	32	64.0	40	81.6	0.052^b^
Marital status					0.770^b^
Married/Common law marriage	26	52.0	24	49.0	
Single/Widowed/Divorced	24	48.0	25	51.0	
Education					0.920^b^
High school/Undergraduate course	25	50.0	25	51.0	
Elementary education	25	50.0	24	49.0	
Occupation					0.310^b^
Retired/Pensioner	36	72.0	41	83.7	
Employee/Self-employed/Homemaker	12	24.0	6	12.2	
Unemployed	2	4.0	2	4.1	
Income					0.520^b^
1 to 3 minimum wages	31	62.0	35	71.4	
4 to 10 minimum wages	14	28.0	9	18.4	
Others (without income or > 10 salaries)	5	10.0	5	10.2	
Religious Practice (yes)	40	80.0	44	89.8	0.280^b^
Diagnosis					0.070^b^
Breast	16	32.0	23	46.9	
Colorectal	16	32.0	3	6.1	
Trachea. bronchus and lung	4	8.0	3	6.1	
Gynecological (uterus and ovary)	4	8.0	8	16.3	
Gastroesophageal	4	8.0	3	6.1	
Pancreas/Biliary Tract	3	6.0	3	6.1	
Prostate	1	2.0	3	6.1	
Other locations	2	4.0	3	6.1	
Metastases					0.510^b^
Hepatic	21	42.0	12	24.5	
Lung and pleura	21	42.0	15	30.6	
Lymph nodes	20	40.0	21	42.9	
Bone	17	34.0	22	44.9	
Peritoneum	12	24.0	14	28.6	
Other locations	22	44.0	17	34.7	
Self-perception of disease stage					0.120^b^
Early	11	22.0	4	8.2	
Advanced	23	46	30	61.2	
Unknown	16	32.0	15	30.6	
Palliative Treatment					0.250^b^
Chemotherapy	46	92.0	43	87.8	
Immunotherapy and hormone therapy	7	14.0	9	18.4	
Palliative Care	6	12.0	1	2.0	
Surgery/Radiotherapy	5	6.0	7	2.0	
Reassessment and follow-up	2	4.0	3	6.1	
Self-perception about the treatment					0.600^b^
Healing	16	32.0	17	34.7	
Palliative	13	26.0	16	32.7	
Unknown	21	42.0	16	32.7	
Performance Status - ECOG					0.970^b^
0 and 1	43	86.0	42	87.7	
2 and 3	7	14.0	7	14.3	
Mental Health Treatments					0.340^b^
Antidepressants	16	32.0	15	30.6	
Sedative-hypnotics	7	14.0	10	20.4	
Psychotherapy	1	2.0	2	7.1	
Mood stabilizers	-	-	1	2.0	
Age (year) ( $\bar{\text{x}}$ ± SD)	56.0 ± 10.7		58.5 ± 13.6		0.310^a^
Children ( $\bar{\text{x}}$ ± SD)	2.4 ± 1.5		2.2 ± 1.7		0.540^a^

$\bar{\text{x}}$ : Mean; SD: Standard deviation;

^a^Mann-Whitney Test;

^b^Fisher exact test.

### Outcomes

**Figure 2:**
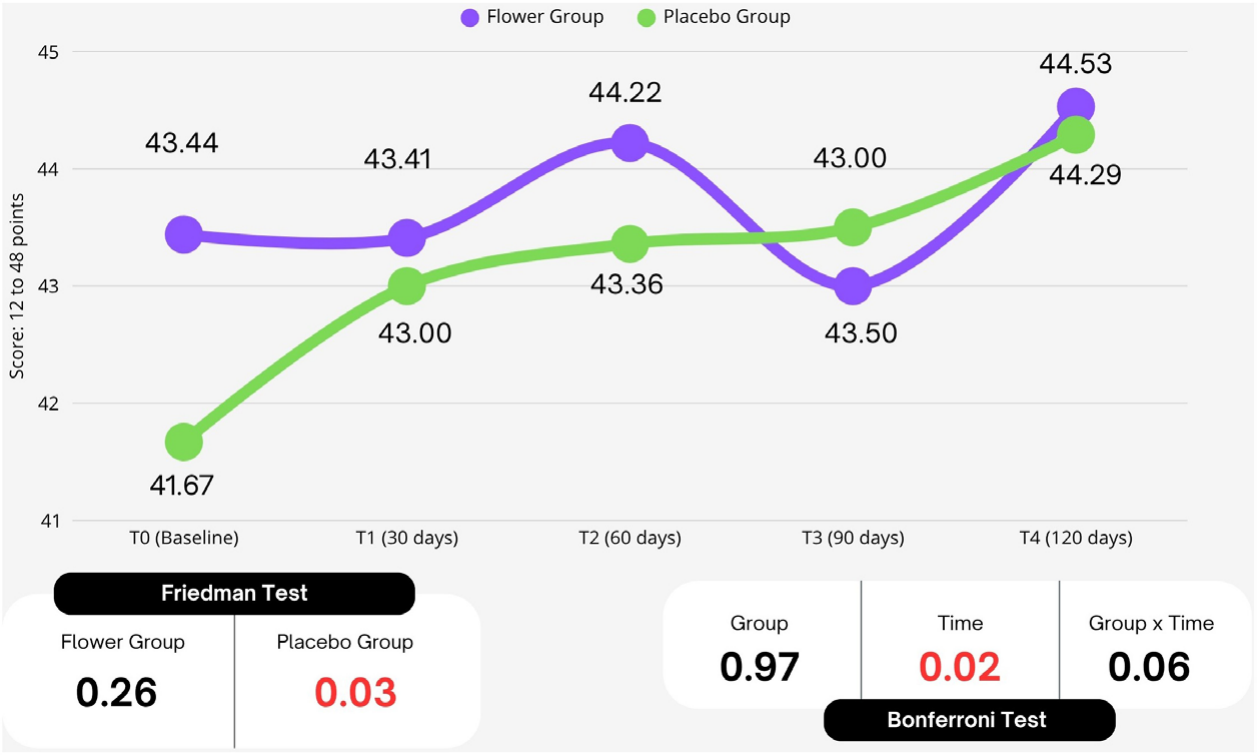
Evaluation of the outcome in the context of hope.

Hope scores remained high from baseline, with slight variation across the segment in both groups. Based on the GLMM statistical model after Bonferroni correction for multiple comparisons, there was an effect of time (p = 0.024), but not between the groups (p = 0.97) (Figure 2).

In the HRQoL, there was a slight improvement in all domains in the FG, but only social well-being (p = 0.022) and additional worries (p = 0.049) showed significance over time, with no difference between the groups (Table 2).

**Table 2:** Analysis of the outcome in Health-Related Quality of Life (FACIT-Pal) – Curitiba, PR, Brazil, 2025.

Group	Evaluation	n	Physical well-being (PWB)			Social well-being (SWB)			Emotional well-being (EWB)			Functional well-being (FWB)		
			Mean	SD	p	Mean	SD	p	Mean	SD	p	Mean	SD	p
Flower	T_0_ (Baseline)	50	22.36	4.77	0.225^1^ 0.168^2^ 0.317^3^	19.86	5.13	0.160^1^ 0.022^2^ 0.174^3^	19.46	4.41	0.229^1^ 0.117^2^ 0.256^3^	18.76	6.04	0.885^1^ 0.710^2^ 0.974^3^
	T_1_ (30 days)	27	22.41	5.55		19.78	5.40		19.93	5.05		19.19	6.22	
	T_2_ (60 days)	18	22.94	5.34		22.11	4.60		21.22	2.62		19.61	5.65	
	T_3_ (90 days)	19	22.74	5.75		20.26	5.76		20.84	3.79		20.33	6.32	
	T_4_ (120 days)	15	23.07	5.51		22.00	5.92		20.53	4.29		20.60	7.32	
Placebo	T_0_ (Baseline)	49	21.06	5.35		18.79	5.33		18.53	4.78		17.90	5.46	
	T_1_ (30 days)	33	21.52	5.52		18.59	5.11		19.00	3.78		18.88	4.17	
	T_2_ (60 days)	25	21.68	4.81		19.75	4.02		18.97	3.66		19.12	4.39	
	T_3_ (90 days)	20	19.40	5.86		19.55	4.62		19.05	3.41		19.05	4.49	
	T_4_ (120 days)	17	22.82	3.47		19.31	4.37		21.29	2.64		20.12	4.37	
			**Additional concerns (PalS)**			**FACT-G**			**TOI**			**FACIT-Pal**		
Flower	T_0_ (Baseline)	50	60.94	10.43	0.433^1^ 0.049^2^ 0.559^3^	80.44	16.29	0.156^1^ 0.323^2^ 0.972^3^	102.07	18.52	0.330^1^ 0.405^2^ 0.788^3^	141.39	25.50	0.230^1^ 0.240^2^ 0.877^3^
	T_1_ (30 days)	27	59.93	13.00		81.30	19.91		101.52	23.25		141.23	32.08	
	T_2_ (60 days)	18	62.89	9.96		85.89	15.83		105.44	18.81		148.78	24.70	
	T_3_ (90 days)	19	62.46	9.63		84.18	18.86		105.53	19.93		146.63	27.79	
	T_4_ (120 days)	15	64.40	11.72		86.20	21.33		108.07	23.53		150.60	32.69	
***Placebo***	T_0_ (Baseline)	49	57.48	10.49		76.28	15.94		96.44	18.77		133.76	25.45	
	T_1_ (30 days)	33	59.91	8.99		77.98	13.20		100.30	15.18		137.89	20.11	
	T_2_ (60 days)	25	60.20	9.07		79.52	11.64		101.00	15.66		139.72	19.57	
	T_3_ (90 days)	20	60.75	7.85		77.05	11.95		99.20	13.27		137.80	18.01	
	T_4_ (120 days)	17	65.41	5.33		83.54	9.88		108.35	10.64		148.96	13.93	

^1-3^p-value based on the GLMM statistical model after Bonferroni test correction for *post hoc* multiple comparisons regarding group^1^,

time^2^,

and group-by-time interaction^3^.

The hope score and HRQoL were positively correlated (p < 0.05) with all domains, subscales, and total score in both groups. These constructs are strongly related, with high levels of hope associated with the different domains of HRQoL (Table 3).

**Table 3:** Correlation between the hope score (HHI) and HRQoL (FACIT-Pal) – Curitiba, PR, Brazil, 2025.

Domains, subscales, and total score	Flower Group (n = 50)	p	Placebo Group (n = 49)	p
	Spearman R		Spearman R	
Physical well-being (PWB)	0.427	0.002	0.367	0.010
Social welll-being (SWB)	0.415	0.003	0.503	0.000
Emotional well-being (EWB)	0.653	0.000	0.496	0.000
Functional well-being (FWB)	0.465	0.001	0.473	0.001
FACT-G	0.606	0.000	0.572	0.000
Additional concerns (PalS)	0.503	0.000	0.694	0.000
TOI	0.512	0.000	0.645	0.000
FACIT-Pal	0.560	0.000	0.671	0.000

Abbreviations: FACT-G, Functional Assessment of Cancer Therapy-General; TOI, Trial Outcome Index; FACIT-Pal, Functional Assessment of Chronic Illness Therapy - Palliative Care.

## DISCUSSION

In this randomized, triple-blind, placebo-controlled clinical trial, BFR did not show a superior effect to placebo on hope and HRQoL in people with advanced cancer on PT.

The absence of a significant difference between the groups can be explained by multiple factors. In the context of palliative care research, it is recognized that systematic follow-up, periodic assessments, and support provided by the research team function as interventions in themselves, producing a placebo effect that reinforces feelings of hope and improves the perception of quality of life^(3,14)^.

A meta-analysis synthesis of multiple cancer types and stages during systemic therapy identified a mean hope score of 35.6^(8)^. Our findings indicate a superiority of approximately 9 points in people with advanced cancer on PT. This is a challenging condition in which these individuals develop coping strategies, visualization, planning, and execution of actions to achieve more favorable outcomes, which are essential in the fight against hopelessness and the promotion of positive thinking, even in the face of advanced disease progression and clinical limitations^(22)^.

The cutoff point establishes that >40 is considered a high hope score in people with cancer, and the clinical relevance of an intervention would have a difference greater than five points^(5)^. It is worth highlighting that, in our findings, the participants already appeared to have high scores at baseline, which limits the possibility of additional gains and characterizes a possible ceiling effect on the hope score^(5)^. Hope is a complex construct, influenced by sociodemographic, clinical, existential, and relational dimensions, which hinders the detection of significant changes from a single intervention; most studies with moderate to small effects have been based on hope theory or advance care planning^(5,23)^.

The scarcity of studies in cancer patients conducted with BFR restricts comparisons. The only available experimental study used a formula (*Gorse, Star of Bethlehem, Gentian, Mimulus, Wild Oat, Honeysuckle and Heather*) and was conducted on Cuban patients; the statistical significance of the FG compared to conventional treatment with antidepressants is questioned due to the high risk of bias^(24)^.

In addition to the high hope score, this research identified inaccurate perceptions about the diagnosis and treatment goals. People with advanced cancer succumb to various cognitive biases and are more likely to believe that the disease is curable and that they will have a longer survival time^(23)^. Thus, it is possible to question whether the imprecision of diagnostic and therapeutic knowledge may have impacted the overvaluation of hope and HRQoL.

Maintaining or improving HRQoL and survival are the goals of the different types of PT. People with advanced cancer are constantly adapting to the chronicity of the disease, experiencing distinct particularities from the initial or terminal phase, undergoing behavioral, psychological, and existential transformations^(2,25)^. This is a phase permeated by internal processes that result in physical and psycho-emotional suffering^(9)^. Frequently, before PT, these patients already presented with declines in different domains of HRQoL, mainly in the physical, emotional, and social domains, which will not always be optimized after treatment^(26)^.

The result of this clinical study differs from international literature, finding higher scores in different domains, subscales, and the total score of the FACIT-Pal^(27)^. The presence of high HRQoL scores at baseline may reflect the ceiling effect, in which participants with better scores in the functional domain reduce the sensitivity for detecting small variations during follow-up, and these variations are not captured as statistically significant. Although clinically significant change threshold estimates are unknown for the FACIT-Pal, a mean difference of less than 5 points has been suggested, given the complexity in detecting robust differences in this population^(28)^.

Even with the limitation of studies in people with cancer, the intervention shows positive results in HRQoL in other populations, as exemplified by the quasi-experimental study, which verified the effect of BFR (*Rescue Remedy®* and *Walnut)* in nursing professionals in Brazil, with significant results on the traumatic stress subscale after three weeks^(29)^. Another formula (*Rescue Remedy®, Red Chestnut, Aspen, Crab Apple, Oak and Olive*), used in a study conducted using mixed methods in Spanish healthcare professionals, indicates significant results in the dimensions of mental health, insomnia, and emotional control^(15)^.

In the therapeutic process with BFR, people may access deep emotional and mental patterns of consciousness, which can cause discomfort or denial, leading them to accept or reject emotional transformations^(11,30)^. In some situations, people may experience a worsening of signs and symptoms, possibly explained by catharsis, in which negative emotions (sadness, anger, fear, or frustration) are initially released, followed by an improvement in emotional well-being^(11)^. This fact can be observed in the oscillation of the regression lines of the hope score, in the domains of social well-being and additional concerns. It is important to consider that the therapeutic effect is not uniform or automatic, a fact that may result in a lack of significance due to the smaller difference between the FG and PG groups^(30)^.

This research identified a high number of participants lost to follow-up (due to insufficient adherence or failure to perceive the effect). In another pragmatic clinical study in Brazil, participants did not identify an expectation of “no improvement” regarding BFR. This fact may influence the perception of positive results in both groups and, coupled with the low participant adherence during the study, reflects the reality of this intervention^(14)^, although a mixed-methods systematic review identifies various barriers regarding the use of NPT, ranging from lack of public awareness and funding to limited evidence and low acceptance among healthcare professionals^(10)^.

The use of BFR in clinical studies^(14,15,16,24,30)^, systematic^(13)^ and scoping^(12)^ reviews differs from what was observed in this study regarding the absence of adverse events. It is noteworthy that a preservative with a low alcohol content was used in the formula composition. However, there are limitations to establishing a causal relationship, since the effects may be related to the clinical characteristics of this population, multiple previous toxicities, use of chemotherapeutic agents, comorbidities, and polypharmacy.

This study has limitations, including a predominantly female sample with breast cancer, which restricts the generalizability of the findings. Other types of cancer and male patients may experience hope and HRQoL differently. Furthermore, the inclusion of participants with psychiatric comorbidities, without subgroup analysis, may have influenced the results. Another relevant point refers to the flower formula. According to Bach’s recommendations, the formulas should be personalized to meet the needs and specificities of each individual. Bach believed that flowers promoted positive emotional states by catalyzing the patient’s internal resources, contributing to the restoration of emotional balance^(11)^.

Another limiting factor is that it was not possible to guarantee strict control over adherence to the recommended dosage. Using it for 120 days may have been excessive and, combined with a delay in perceiving the subtle effects of BFR, may have contributed to poor adherence. The use of brandy (an alcoholic preservative), even in low concentrations, may have been responsible for the reported mild adverse events.

## CONCLUSION

Bach Flower Therapy, in the tested formulation (*Cherry Plum, Crab Apple, Gorse, Mimulus, Mustard, Olive, Sweet Chestnut, White Chestnut and Wild Oat*), did not show a superior effect compared to placebo on hope or health-related quality of life in patients with advanced cancer. Although both groups showed a slight increase in the scores assessed, this result may reflect the placebo effect and the clinical attention received. Associated with the reported mild adverse events, the findings also suggest caution in the use of alcoholic preservatives in this population.

These findings reinforce the need for robust clinical trials, with personalized interventions, better adherence monitoring, and the use of sensitive psychometric measures. For Nursing, the study contributes to strengthening evidence-based practice in palliative care and broadening reflection on the role of non-pharmacological therapies.

## DATA AVAILABILITY

The entire dataset supporting the results of this study was published in the article itself.
