# Supplementary material for: The effect of Bach flower remedies on hope and quality of life in people with advanced cancer: a triple-blind randomized clinical trial
Source: Rev Esc Enferm USP. 2026 Jul 20;60:e20250451. doi: 10.1590/1980-220X-REEUSP-2025-0451en (PMC13390996; doi:10.1590/1980-220X-REEUSP-2025-0451en)
Supplement: Translation

# Translation: Efeito do floral de Bach na esperança e qualidade de vida de pessoas com câncer avançado: ensaio clínico randomizado triplo cego

**DOI:** 10.1590/1980-220X-REEUSP-2025-0451pt

**Authors:** Leonel dos Santos Silva, Amanda Gomes de Miranda, Larissa Marcondes, Ruth Natalia Teresa Turrini, Paulo Ricardo Bittencourt Guimarães, Maria de Fátima Mantovani, Luciana de Alcantara Nogueira, Luciana Puchalski Kalinke

**Affiliations:** 1 Universidade Federal do Paraná, Programa de Pós-Graduação em Enfermagem, Curitiba, PR, Brasil.; 2 Universidade de São Paulo, Escola de Enfermagem, Departamento de Enfermagem Médico-Cirúrgica, São Paulo, SP, Brasil.; 3 Universidade Federal do Paraná, Departamento de Estatística, Curitiba, PR, Brasil.

**Keywords:** Essências Florais, Esperança, Qualidade de Vida, Oncologia Integrativa, Cuidados Paliativos

## Abstract

**Objetivo:**

Avaliar o efeito da Terapia Floral de Bach na esperança e qualidade de vida de pessoas com câncer avançado.

**Método:**

Ensaio clínico randomizado, triplo cego (1:1), em hospital público de alta complexidade, região Sul do Brasil. Noventa e nove participantes foram alocados no grupo intervenção (solução com nove essências florais) ou grupo placebo (solução Hydro-brandy). Ambos utilizaram quatro gotas, via oral, quatro vezes ao dia, durante 120 dias, com seguimento mensal. Esperança mensurada pelo *Herth Hope Index*, qualidade de vida pelo *Functional Assessment of Chronic Illness Therapy* – *Palliative Care*.

**Resultados:**

No grupo floral (n = 50), a esperança variou de 43,44/48 para 44,53/48 e 41,67/48 para 44,29/48 (placebo /n = 49), aumento significativo no tempo (p = 0,024), sem diferença entre grupos (p = 0,977). A qualidade de vida variou de 141,39/184 para 150,60/184 (grupo floral), e 133,76/184 para 148,96/184 (placebo), sem significância para grupos (p = 0,230) ou tempo (p = 0,240).

**Conclusão:**

Embora sem significância estatística, houve discreto aumento em esperança e qualidade de vida, ressaltando a importância das terapias não farmacológicas na prática baseada em evidências em cuidados paliativos.

**Registo Brasileiro de Ensaio Clínico:**

RBR-8q6z6kq.

## INTRODUÇÃO

Entre a restrita visão dicotômica do curável e incurável, a doença avançada está intrinsecamente associada ao sofrimento e enfrentamento. Nos estágios avançados do câncer, o Tratamento Paliativo (TP), Cuidados Paliativos (CP) e de Suporte buscam melhorar a Qualidade de Vida Relacionada à Saúde (QVRS) e prolongar a sobrevida^(1,2)^.

Independente do estágio da doença ou objetivos terapêuticos, o CP precoce é indicado por ter abordagem centrada na pessoa e seus familiares, uma visão disruptiva do modelo limitada a doença e por cuidar das dimensões biopsicosocioespirituais para manter a sobrevida com qualidade^(1,2,3)^. Muitas pessoas estão em TP sem a integração precoce com abordagens de CP, por mais que compartilhem objetivos para beneficiar pacientes em condições clínicas elegíveis.

No câncer avançado ou incurável, diferentes modalidades de TP estão indicadas, sejam elas sistêmicas (quimioterapia, hormonioterapia, imunoterapia e terapia direcionada) ou localizadas (radioterapia e cirurgia), direcionadas ao controle da progressão das metástases. São terapêuticas complexas, manejadas em unidades ambulatoriais ou internações por equipes transdisciplinares, que devem ser ofertadas de maneira oportuna e concomitante com abordagem de CP^(2)^.

No contexto paliativo, a produção cientifica é amplamente direcionada a investigar e otimizar a QVRS, compreendida como a percepção da pessoa sobre o impacto da doença e tratamento na multidimensionalidade física, funcional, social, psicoemocional, existencial e de finitude^(1,3,4)^. No entanto, nas pessoas com câncer avançado, a esperança permanece subinvestigada, sendo destacada em apenas 4% das publicações^(3)^. A esperança coexiste com a saúde-doença-cuidado crônico, é um mecanismo de enfrentamento que precisa ser avaliado e promovido^(5)^.

A esperança é a força motriz para sobrevivência, não limitada por traços, conceitos lineares ou determinada externamente, entendida como “uma força vital e dinâmica […] caracterizada por uma expectativa confiante…”^(6)^, ela é “um atributo motivacional e cognitivo […] para iniciar e manter a ação em direção ao alcance da meta”^(7)^. A esperança está relacionada com fatores sociodemográficos, clínicos, terapêuticos, QVRS, questões existenciais, apoio social, psicologia positiva, resiliência, *coping*, entre outros^(5,8,9)^.

A Psycho-Oncology e Oncologia Integrativa continuamente dedicam-se à busca por intervenções não invasivas e não farmacológicas para a saúde humana com embasamento científico, segurança e eficácia, aqui denominados de Terapias não Farmacológicas (TNF), para oferecer o cuidado holístico^(4)^. Dentre as 604 diferentes TNF classificadas, as Essências ou Terapia Floral de Bach (TFB) foi desenvolvida na Inglaterra entre os anos de 1928 e 1935, pelo médico homeopata Edward Bach^(10)^. São soluções altamente diluídas em água, extraídas de plantas e flores silvestres, que se destinam ao equilíbrio psicoemocional, por atuarem em níveis sutis de harmonização interna da pessoa e ambiente^(11,12)^.

Por não estar claro se existem resquícios farmacológicos, não existe um consenso sobre o mecanismo de ação da TFB. Uma das hipóteses é que a solução ultra diluída contenha nanopartículas com informações/energia sutil das flores e tenha uma ação psicomoduladora no consciente e inconsciente, para reequilibrar estados emocionais^(11,13,14,15)^.

Mesmo diante das incertezas relacionadas ao mecanismo de ação da TFB, ela é reconhecida pela Organização Mundial de Saúde (1976), autorizada pela Política Nacional de Práticas Integrativas e Complementares (2018), com respaldo do Conselho Federal de Enfermagem. É utilizada na sociedade com expectativa de algum resultado; consequentemente, alguns estudos destacam seus efeitos no transtorno do déficit de atenção e hiperatividade^(13)^; para condições que interferem no corpo e mente^(12)^; na redução do estresse, ansiedade, compulsão alimentar, frequência cardíaca^(16)^; e na saúde mental, insônia e gestão das emoções^(15)^.

Apesar do crescente interesse pela Oncologia integrativa, são escassos os ensaios clínicos randomizados que avaliam o efeito da Terapia Floral de Bach em câncer avançado, com foco em esperança e qualidade de vida. Neste contexto, este estudo teve como objetivo avaliar o efeito da Terapia Floral de Bach na esperança e qualidade de vida de pessoas com câncer avançado.

## MÉTODO

### Desenho do Estudo

Ensaio clinico randomizado, triplo cego, controlado por placebo, conduzido no ambulatório de Oncologia-Hematologia de um hospital público na região Sul do Brasil. Duas diretrizes foram seguidas ao reportar este ensaio: *Consolidated Standards of Reporting Trials* (CONSORT) e sua extensão para *Nonpharmacologic Treatments* (NPT)^(17)^.

### Participantes e Amostra

Dos participantes voluntários, foram incluídos: adultos (≥ 18 anos) com câncer avançado (estágio IV), classificados na escala *performance status da Eastern Cooperative Oncology Group* (PS-ECOG) de 0-3. Excluíram-se os casos de doenças linfoproliferativas, pacientes exclusivamente em Cuidados Paliativos e aqueles sem condições de comunicação verbal e/ou escrita.

O tamanho da amostra foi calculado no *software* GPower (v 3.1.9.7) para modelo longitudinal (2 grupos e 5 medidas), assumindo f = 0,25, α = 5% e poder de 95%. Considerando perdas previstas, planejou-se recrutar 125 pacientes. O recrutamento foi realizado de outubro de 2022 a fevereiro de 2024 e avaliação de seguimento até junho de 2024.

### Aspectos Éticos

O estudo foi aprovado pelo comitê de ética em pesquisa institucional com parecer n° 5.204.355 e homologado no Registros Brasileiros de Ensaios Clínicos Randomizados (ReBEC) sob parecer n° RBR-8q6z6kq. Todos os participantes receberam esclarecimento verbal, escrito e assinaram o consentimento informado antes da inclusão na pesquisa.

### Intervenção

Todos os frascos eram idênticos, de vidro âmbar estéreis, capacidade para 30 mL, com tampa furada, lacre de segurança, conta-gotas com bulbo branco e cânula de vidro de 75 mm. As soluções preparadas poderiam conter a fórmula floral (GF) ou a solução placebo (GP). Todos eram rotulados de forma semelhante “Pesquisa Floral” e instruções de uso, com diferença apenas na etiqueta numérica e sequencial.

As soluções foram preparadas em farmácia especializada, em frascos idênticos de 30ml. Cada frasco continha um volume para 30 dias de uso, com margem adicional de 30%. O Grupo floral recebeu hidro-brandy acrescido de nove essências de florais de Bach, e o placebo apenas o hidro-brandy. Ambos os grupos ingeriram quatro gotas, quatro vezes ao dia, por 120 dias. Mensagens via WhatsApp® foram enviadas nas primeiras oito semanas para estimular a adesão.

A solução hidro-brandy era composta de 21 mL de água mineral (marca Crystal®, densidade 1,0 e pH a 25ºC = 8,71) + 9 mL de conservante *brandy* (marca Osborne®, 100% destilado de vinho e 36% vol. alc.). A solução floral era composta por 2 gotas da essência-mãe floral (com 400 partes de conhaque orgânico em uma parte de essência mãe da Healing Herbs® proveniente da Inglaterra). A fórmula (e as principais indicações) era composta por ***Cherry Plum*** (*Prunus cerasifera*, autocontrole emocional), ***Crab Apple*** (*Malus pumila,* aceitação e autoestima), ***Gorse*** (*Ulex europaeus,* fé e esperança), ***Mimulus*** (*Mimulus guttatus*, aceitação), ***Mustard*** (*Sinapis arvensis*, melancolia), ***Olive*** (*Olea europaea,* exaustão fisica), ***Sweet Chestnut*** (*Castanea sativa*, enfrentamento de adversidades), ***White Chestnut*** (*Aesculus hippocastanum*, discernimento) e ***Wild Oat*** (*Bromus ramosus,* propósito e sentido)^(11)^.

Ambos os grupos continuaram recebendo o tratamento oncológico com intenção paliativa, seja ele sistêmico ou localizado conforme a condição clínica de cada indivíduo.

### Desfechos

O desfecho primário foi avaliado pelo Índice de Esperança de Herth (HHI), instrumento mais sumarizado em revisões sistemáticas sobre o constructo em oncologia^(8,9)^. Trata-se de uma avaliação breve do autorrelato de esperança composto por 12 itens em uma escala Likert. O escore total varia de 12 à 48, é a somatória do intervalo possível, quanto mais elevado denota maior esperança^(7)^. Pode ser classificado em três níveis de esperança com base em suas pontuações: baixo (12–23), médio (24–35) ou alto (36–48). A consistência interna do instrumento estimada pelo α de Cronbach na versão original foi de 0,97 (excelente)^(7)^, 0,834 (boa) em sua validação e adaptação cultural no Brasil^(18)^.

O desfecho secundário avaliou a QVRS, com o instrumento de autorrelato *Functional Assessment of Chronic Illness Therapy - Palliative Care* (FACIT-Pal, versão 4). A FACIT-Pal é composta pelas quatro dimensões de bem-estar da *Functional Assessment of Cancer Therapy* (FACT-G): físico (PWB: 7 itens, 0-28 pontos); social/familiar (SWB: 7 itens, 0-28 pontos), emocional (EWB: 6 itens, 0-24 pontos), funcional (FWB 7 itens, 0-28 pontos) e uma subescala de domínio paliativo (PalS 19 itens, 0-76 pontos)^(19)^. Os itens são pontuados no formato likert de 0 (nem um pouco) a 4 (muito) pontos; maiores pontuações refletem melhor QVRS. As subescalas resultam no Índice de Resultado do Teste (*Trial Outcome Index* – TOI: PWB + FWB + PalS = 0 a 132 pontos) e no escore total (0-184pontos)^(19)^. A confiabilidade da consistência interna do α de Cronbach varia de boa a excelente na versão original das subescalas (FACT-G: 0,89; TOI: 0,91e FACIT-Pal: 0, 93)^(19)^ e na sua tradução para português (FACT-G: 0,91)^(20)^.

Eventos adversos foram monitorados em todas as visitas, classificados por gravidade e relação causal. Eventos graves implicaram em descontinuação da intervenção, mantendo análise de intenção de tratar.

### Coleta de Dados

A coleta de dados foi realizada no REDCap®. Na inclusão dos participantes, foram aplicados os instrumentos, de forma presencial, para obtenção de: dados sociodemográficos e clínicos; esperança e QVRS (baseline, T_0_). Nas avaliações de seguimento, a cada ± 30 dias, foram aplicados os instrumentos de esperança e QVRS (T_1_ a T_4_ – 120 dias), seja de forma presencial, por telefone ou envio de link para o participante.

Foram descontinuados os participantes ausentes ± 30 dias após o T_0_ ou as últimas avaliações de seguimento (T_1_ a T_4_), que não fizeram adesão superior a 70% do conteúdo do frasco conforme relato verbal, ou na vigência de qualquer evento adverso relacionado.

### Randomização e Alocação

Uma sequência de alocação aleatória com 150 números (1 para intervenção e 2 para placebo) foi gerada no site (https://ctrandomization.cancer.gov/tool/), preservado o equilíbrio de alocação 1:1 entre os grupos, procedimento realizado por uma pesquisadora não cega (sem contato com os participantes ou pesquisador de campo). A randomização foi entregue para a farmacêutica (sem contato com os participantes ou pesquisador de campo) responsável pela manipulação, esses códigos numéricos foram utilizados para identificar os frascos, determinar o conteúdo (TFB ou placebo) e assegurar o mascaramento de alocação (participantes e pesquisador de campo).

### Mascaramento

Foi mantido o mascaramento triplo (participantes, pesquisador de campo e estatístico) até o término do estudo. Uma pesquisadora não cega realizou a randomização e a farmacêutica fez a manipulação, ambas não mantinham contato com os participantes, o pesquisador de campo e o estatístico. A farmácia de manipulação era distante do ambulatório de coleta de dados. O pesquisador de campo mantinha contato com a equipe de dispensação e foi responsável pela coleta de dados. Após o término da coleta, o pesquisador de campo encaminhou os dados para a pesquisadora que fez a randomização, a qual, de posse da randomização, organizou os dados em grupos A e B e enviou-os ao estatístico. Após as análises, a pesquisadora não cega esclareceu qual dos grupos era o floral ou placebo.

### Análise dos Dados

Os dados capturados foram exportados do REDCap® e analisados com o uso do *software* SPSS® 20. Na verificação da homogeneidade dos grupos, foram utilizados os testes Qui-quadrado ou Exato de Fisher para as variáveis qualitativas e o teste t de Student para variáveis quantitativas.

As análises seguiram o princípio da intenção de tratar, para minimizar o viés de seleção após a randomização, associado ao Modelo Linear Misto Generalizado (GLMM), devido à necessidade de incorporar todas as avaliações disponíveis de cada participante ao longo do tempo até o momento da descontinuação, pelo princípio da máxima verossimilhança^(21)^. Foram adotados uma matriz de covariância não estruturada e o Critério de Informação de Akaike (AIC) para seleção de modelo. O nível de significância foi fixado em 5%, com correção de Bonferroni para comparações múltiplas.

As correlações entre os escores de esperança e FACIT-Pal foram verificadas pelo do coeficiente Spearman. O nível de significância foi estabelecido em 5%.

## RESULTADO

Dos 125 pacientes elegíveis, 99 foram randomizados (GF = 50, GP = 49). As perdas foram maiores nas fases iniciais, sobretudo por não adesão ou óbito. Ao final do segmento, foram analisados 15 participantes no GF e 17 no GP (Figura 1). Os óbitos foram decorrentes da evolução da doença ou complicações relacionadas ao tratamento, sem relação com a intervenção.

**Figura 1:** Fluxograma dos participantes.

### Características dos Participantes

Os grupos foram homogêneos quanto às variáveis sociodemográficas, clínicas e terapêuticas (p > 0,05) (Tabela 1).

**Tabela 1:** Características dos participantes do Grupo Floral (GF) e Grupo Placebo (GP) – Curitiba, PR, Brasil, 2025.

	Grupo floral (n = 50)		Grupo placebo (n = 49)		*p*
	n	(%)	n	(%)	
Mulheres	32	64,0	40	81,6	0.052^b^
Situação Conjugal					0.770^b^
Casadas/União estável	26	52,0	24	49,0	
Solteira/Viúva/Divorciada	24	48,0	25	51,0	
Educação					0.920^b^
Ensino médio/Graduação	25	50,0	25	51,0	
Ensino fundamental	25	50,0	24	49,0	
Ocupação					0.310^b^
Aposentado/Pensionista	36	72,0	41	83,7	
Empregado/Autônomo/Do lar	12	24,0	6	12,2	
Desempregado	2	4,0	2	4,1	
Rendimento					0.520^b^
1 a 3 salários-mínimos	31	62,0	35	71,4	
4 a 10 salários-mínimos	14	28,0	9	18,4	
Outros (sem renda ou > 10 salários)	5	10,0	5	10,2	
Prática Religiosa (sim) Diagnóstico	40	80,0	44	89,8	0.280^b^
					0.070^b^
Mama	16	32,0	23	46,9	
Colorretal	16	32,0	3	6,1	
Traqueia, brônquio e pulmão	4	8,0	3	6,1	
Ginecológico (útero e ovário)	4	8,0	8	16,3	
Gastresofágico	4	8,0	3	6,1	
Pâncreas/Vias Biliares	3	6,0	3	6,1	
Próstata	1	2,0	3	6,1	
Outras localizações	2	4,0	3	6,1	
Metástases					0.510^b^
Hepática	21	42,0	12	24,5	
Pulmão e pleura	21	42,0	15	30,6	
Linfonodos	20	40,0	21	42,9	
Óssea	17	34,0	22	44,9	
Peritônio	12	24,0	14	28,6	
Outras localizações	22	44,0	17	34,7	
Autopercepção do estágio da doença					0.120^b^
Inicial	11	22,0	4	8,2	
Avançado	23	46	30	61,2	
Desconhece	16	32,0	15	30,6	
Tratamento Paliativo					0.250^b^
Quimioterapia	46	92,0	43	87,8	
Imunoterapia e hormonioterapia	7	14,0	9	18,4	
Cuidados Paliativos	6	12,0	1	2,0	
Cirurgia/Radioterapia	5	6,0	7	2,0	
Reavaliação e acompanhamento	2	4,0	3	6,1	
Autopercepção sobre o tratamento					0.600^b^
Curativo	16	32,0	17	34,7	
Paliativo	13	26,0	16	32,7	
Desconhece	21	42,0	16	32,7	
Performance Status - ECOG					0.970^b^
0 e 1	43	86,0	42	87,7	
2 e 3	7	14,0	7	14,3	
Tratamentos em Saúde Mental					0.340^b^
Antidepressivos	16	32,0	15	30,6	
Sedativos-hipnóticos	7	14,0	10	20,4	
Psicoterapia	1	2,0	2	7,1	
Estabilizadores de humor	-	-	1	2,0	
Idade (anos) ( $\bar{\text{x}}$ ± DP)	56,0 ± 10,7		58,5 ± 13,6		0,310^a^
Filhos ( $\bar{\text{x}}$ ±DP)	2,4 ± 1,5		2,2 ± 1,7		0,540^a^

$\bar{\text{x}}$ : Média; SD: Desvio padrão;

^a^Mann-Whitney Test;

^b^Fisher exact test.

### Desfechos

A esperança apresentou escores elevados desde o baseline, com discreta variação ao longo do segmento em ambos os grupos. Com base no modelo estatístico GLMM após a correção do teste de Bonferroni para comparações múltiplas, houve efeito do tempo (p = 0,024), porém não entre os grupos (p = 0,97) (Figura 2).

**Figura 2:** Avaliação do desfecho na esperança.

Na QVRS, houve melhora discreta em todos os domínios no GF, mas apenas o bem-estar social (p = 0,022) e preocupações adicionais (p = 0,049) mostraram significância ao longo do tempo, sem diferença entre os grupos (Tabela 2).

**Tabela 2:** Análise do desfecho na Qualidade de Vida Relacionada à Saúde (FACIT-Pal) – Curitiba, PR, Brasil, 2025.

Grupo	Avaliação	n	Bem-estar físico (PWB)			Bem-estar social (SWB)			Bem-estar emocional (EWB)			Bem-estar funcional (FWB)		
			Média	DP	p	Média	DP	p	Média	DP	p	Média	DP	p
Floral	T_0_ (Baseline)	50	22.36	4.77	0,225^1^ 0,168^2^ 0,317^3^	19.86	5.13	0,160^1^ 0,022^2^ 0,174^3^	19.46	4.41	0,229^1^ 0,117^2^ 0,256^3^	18.76	6.04	0,885^1^ 0,710^2^ 0,974^3^
	T_1_ (30 dias)	27	22.41	5.55		19.78	5.40		19.93	5.05		19.19	6.22	
	T_2_ (60 dias)	18	22.94	5.34		22.11	4.60		21.22	2.62		19.61	5.65	
	T_3_ (90 dias)	19	22.74	5.75		20.26	5.76		20.84	3.79		20.33	6.32	
	T_4_ (120 dias)	15	23.07	5.51		22.00	5.92		20.53	4.29		20.60	7.32	
Placebo	T_0_ (Baseline)	49	21.06	5.35		18.79	5.33		18.53	4.78		17.90	5.46	
	T_1_ (30 dias)	33	21.52	5.52		18.59	5.11		19.00	3.78		18.88	4.17	
	T_2_ (60 dias)	25	21.68	4.81		19.75	4.02		18.97	3.66		19.12	4.39	
	T_3_ (90 dias)	20	19.40	5.86		19.55	4.62		19.05	3.41		19.05	4.49	
	T_4_ (120 dias)	17	22.82	3.47		19.31	4.37		21.29	2.64		20.12	4.37	
			**Preocupações adicionais (PalS)**			**FACT-G**			**TOI**			**FACIT-Pal**		
Floral	T_0_ (Baseline)	50	60.94	10.43	0,433^1^ 0,049^2^ 0,559^3^	80.44	16.29	0,156^1^ 0,323^2^ 0,972^3^	102.07	18.52	0,330^1^ 0,405^2^ 0,788^3^	141.39	25.50	0,230^1^ 0,240^2^ 0,877^3^
	T_1_ (30 dias)	27	59.93	13.00		81.30	19.91		101.52	23.25		141.23	32.08	
	T_2_ (60 dias)	18	62.89	9.96		85.89	15.83		105.44	18.81		148.78	24.70	
	T_3_ (90 dias)	19	62.46	9.63		84.18	18.86		105.53	19.93		146.63	27.79	
	T_4_ (120 dias)	15	64.40	11.72		86.20	21.33		108.07	23.53		150.60	32.69	
Placebo	T_0_ (Baseline)	49	57.48	10.49		76.28	15.94		96.44	18.77		133.76	25.45	
	T_1_ (30 dias)	33	59.91	8.99		77.98	13.20		100.30	15.18		137.89	20.11	
	T_2_ (60 dias)	25	60.20	9.07		79.52	11.64		101.00	15.66		139.72	19.57	
	T_3_ (90 dias)	20	60.75	7.85		77.05	11.95		99.20	13.27		137.80	18.01	
	T_4_ (120 dias)	17	65.41	5.33		83.54	9.88		108.35	10.64		148.96	13.93	

^1-3^Valor de p com base no modelo estatístico GLMM após a correção do Teste de Bonferroni para comparações múltiplas pós-hoc em relação ao grupo^1^,

tempo^2^

e grupo versus tempo^3^.

O escore de esperança e a QVRS correlacionaram-se positivamente (p < 0,05) com todos os domínios, subescalas e escore total em ambos os grupos. Esses construtos estão fortemente relacionados, com níveis elevados de esperança associados aos diferentes domínios da QVRS (Tabela 3).

**Tabela 3:** Correlação entre o escore de esperança (HHI) e QRVS (FACIT-Pal) – Curitiba, PR, Brasil, 2025.

Domínios, subescalas e escore total	Grupo floral (n = 50)	p	Grupo placebo (n = 49)	p
	Spearman R		Spearman R	
Bem-estar físico (PWB)	0,427	0,002	0,367	0,010
Bem-estar social (SWB)	0,415	0,003	0,503	0,000
Bem-estar emocional (EWB)	0,653	0,000	0,496	0,000
Bem-estar funcional (FWB)	0,465	0,001	0,473	0,001
FACT-G	0,606	0,000	0,572	0,000
Preocupações adicionais (PalS)	0,503	0,000	0,694	0,000
TOI	0,512	0,000	0,645	0,000
FACIT-Pal	0,560	0,000	0,671	0,000

Abreviações: FACT-G, Functional Assessment of Cancer Therapy-General; TOI, Trial Outcome Index; FACIT-Pal, Functional Assessment of Chronic Illness Therapy - Palliative Care.

## DISCUSSÃO

Neste ensaio clínico randomizado, triplo-cego e controlado por placebo, a TFB não apresentou efeito superior ao placebo sobre a esperança e QVRS em pessoas com câncer avançado em TP.

A ausência de diferença significativa entre os grupos pode ser explicada por múltiplos fatores. No contexto da pesquisa em cuidados paliativos, é reconhecido que o acompanhamento sistemático, as avaliações periódicas e o suporte oferecido pela equipe de pesquisa funcionam como intervenções em si, produzindo efeito placebo, que reforçam sentimentos de esperança e melhoram a percepção de qualidade de vida^(3,14)^.

A síntese de uma metanálise com múltiplos tipos e estágios de câncer durante a terapia sistêmica identificou que 35,6 é a média do escore de esperança^(8)^. Nossos achados apontam uma superioridade de aproximadamente 9 pontos em pessoas com câncer avançado em TP. Trata-se de uma condição desafiadora em que essas pessoas desenvolvem estratégias de enfrentamento, visualização, planejamento e execução de ações para alcançarem resultados mais favoráveis, que são essenciais na luta contra a desesperança e promoção de pensamentos positivos, mesmo em face da progressão da doença avançada e limitações clínicas^(22)^.

O ponto de corte estabelece que >40 é considerado escore elevado de esperança em pessoas com câncer e a relevância clínica de uma intervenção teria uma diferença superior a cinco pontos^(5)^. Convém destacar que, em nossos achados, os participantes já aparentavam escores elevados no baseline, o que limita a possibilidade de ganhos adicionais e caracteriza um possível efeito teto no escore de esperança^(5)^. A esperança é um constructo complexo, influenciado por dimensões sociodemográficas, clínicas, existenciais e relacionais, o que dificulta a detecção de mudanças significativas a partir de uma única intervenção; a maioria dos estudos com efeitos moderados a pequenos foram baseados na teoria da esperança ou no planejamento antecipado de cuidados^(5,23)^.

A escassez de estudos em pacientes com câncer conduzidos com a TFB limita as comparações. Um único estudo experimental disponível utilizou uma fórmula (*Gorse, Star of Bethlehem, Gentian, Mimulus, Wild Oat, Honeysuckle e Heather*) e foi conduzido em pacientes cubanos; a significância estatística no GF em comparação ao tratamento convencional com antidepressivos é questionada devido ao alto risco de viés^(24)^.

Além do elevado escore de esperança, a presente pesquisa identificou percepções imprecisas sobre o diagnóstico e os objetivos do tratamento. Pessoas com câncer avançado sucumbem a diversos vieses cognitivos e estão mais propensas a acreditar que a doença é curável e que terão maior sobrevida^(23)^. Assim, é possível questionar se a imprecisão do conhecimento diagnóstico e terapêutico pode ter impactado a supervalorização da esperança e da QVRS.

Manter ou melhorar a QVRS e sobrevivência são os objetivos das diferentes modalidades de TP. As pessoas com câncer avançado estão em constante adequação à cronicidade da doença, vivenciam particularidades distintas da fase inicial ou da terminalidade, perpassam por uma transformação comportamental, psicológica e existencial^(2,25)^. Trata-se de uma fase permeada por processos internos que resultam em sofrimento físico e psicoemocional^(9)^. Frequentemente, antes do TP esses pacientes já apresentavam declínio em diferentes domínios da QVRS, principalmente no físico, emocional e social, os quais nem sempre serão otimizados após o tratamento^(26)^.

O resultado deste estudo clínico diverge da literatura internacional, ao encontrar escores superiores em diferentes domínios, subescalas e total do FACIT-Pal^(27)^. A presença de escores elevados de QVRS no baseline pode refletir o efeito teto, no qual os participantes com melhores pontuações no domínio funcional reduzem a sensibilidade para detecção de pequenas variações no seguimento e estas não são captadas como significância estatística. Embora as estimativas limiares de mudança clinicamente significativas sejam desconhecidas para o FACIT-Pal, foi sugerida uma diferença média inferior a 5 pontos, dada a complexidade em detectar diferenças robustas nesta população^(28)^.

Mesmo com a limitação de estudos em pessoas com câncer, a intervenção apresenta resultados positivos na QVRS em outras populações, a exemplo do estudo quase-experimental, que verificou o efeito da TFB (*Rescue Remedy*® e *Walnut*) em profissionais de enfermagem no Brasil, com resultados significativos na subescala de estresse traumático após três semanas^(29)^. Outra fórmula (*Rescue Remedy*®, *Red Chestnut*, *Aspen, Crab Apple, Oak e Olive*), utilizada no estudo de métodos mistos em profissionais de saúde espanhóis, aponta resultados significativos nas dimensões de saúde mental, insônia e controle emocional^(15)^.

No processo terapêutico com TFB, as pessoas possivelmente acessam padrões emocionais e mentais profundos da consciência, os quais podem causar desconforto ou negação, o que faz com que elas aceitem ou rejeitem as transformações emocionais^(11,30)^. Em algumas situações, as pessoas podem apresentar piora de sinais e sintomas, fato possivelmente explicado pela catarse, em que ocorre inicialmente a liberação de emoções negativas (tristeza, raiva, medo ou frustração), para posterior melhora do bem-estar emocional^(11)^. Este fato pode se ser percebido na oscilação das retas de regressão do escore de esperança, nos domínio bem-estar social e preocupações adicionais. É importante considerar que o efeito terapêutico não é uniforme ou automático, fato que pode resultar na ausência de significância devido a menor diferença entre os GF e GP^(30)^.

Nesta pesquisa, foi identificado elevado número de perdas de seguimento de participantes (adesão insuficiente ou não percepção do efeito). Em outro estudo clínico pragmático, no Brasil, não foi identificada entre os participantes a expectativa de “nenhuma melhora” sobre a TFB. Este fato pode influenciar a percepção de resultados positivos em ambos os grupos e, associado a baixa adesão dos participantes durante o estudo, reflete a realidade dessa intervenção^(14)^, embora uma revisão sistemática de métodos mistos identifique diferentes barreiras quanto ao uso das TNF, desde o desconhecimento pela população, ausência de financiamento, escassa evidência e baixa receptividade dos profissionais de saúde^(10)^.

O uso da TFB em estudos clínicos^(14,15,16,24,30)^, revisão sistemática^(13)^ e escopo^(12)^ difere do observado neste estudo quanto à ausência de eventos adversos. Destaca-se que na composição da fórmula foi utilizado um conservante com baixo teor alcoólico. Entretanto, existem limitações para estabelecer uma relação causal, uma vez que os efeitos podem estar relacionados às características clínicas desta população, múltiplas toxicidades prévias, uso de quimioterápicos, comorbidades e uso de polifarmácia.

Este estudo apresenta limitações, incluindo uma amostra predominantemente feminina com câncer de mama, o que restringe a generalização dos achados. Outros tipos de câncer e pacientes do sexo masculino podem experimentar esperança e QVRS de maneira diferente. Além disso, a inclusão de participantes com comorbidades psiquiátricas, sem análise por subgrupo, pode ter influenciado os resultados. Outro ponto relevante refere-se à fórmula floral. De acordo com as recomendações de Bach, as fórmulas devem ser personalizadas para atender às necessidades e especificidades de cada indivíduo. Bach acreditava que as flores promoviam estados emocionais positivos ao catalisar os recursos internos do paciente, contribuindo para a restauração do equilíbrio emocional^(11)^.

Outro ponto limitante é que não foi possível garantir um controle rigoroso da adesão à dosagem recomendada. A utilização por 120 dias pode ter sido excessiva e, associada ao atraso na percepção sutil do efeito da TFB, pode ter contribuído para a baixa adesão. O uso de brandy (conservante alcoólico), mesmo em baixa concentração, pode ter sido responsável pelos eventos adversos leves relatados.

## CONCLUSÃO

A Terapia Floral de Bach, na formulação testada (*Cherry Plum, Crab Apple, Gorse, Mimulus, Mustard, Olive, Sweet Chestnut, White Chestnut e Wild Oat*), não apresentou efeito superior ao placebo sobre a esperança ou a qualidade de vida relacionada à saúde em pacientes com câncer avançado. Apesar de ambos os grupos terem mostrado discreto aumento nos escores avaliados, tal resultado pode refletir o efeito placebo e a atenção clínica recebida. Associados aos eventos adversos leves relatados, os achados também sugerem cautela no uso de conservantes alcoólicos nessa população.

Estes achados reforçam a necessidade de ensaios clínicos robustos, com intervenções personalizadas, melhor monitoramento da adesão e uso de medidas psicométricas sensíveis. Para a Enfermagem, o estudo contribui para fortalecer a prática baseada em evidências nos cuidados paliativos e ampliar a reflexão sobre o papel das terapias não farmacológicas.

## DISPONIBILIDADE DE DADOS

Todo o conjunto de dados que dá suporte aos resultados deste estudo foi publicado no próprio artigo.

# Translation: El efecto de las flores de Bach en la esperanza y la calidad de vida de las personas con cáncer avanzado: ensayo clínico aleatorizado triple ciego

**DOI:** 10.1590/1980-220X-REEUSP-2025-0451es

**Authors:** Leonel dos Santos Silva, Amanda Gomes de Miranda, Larissa Marcondes, Ruth Natalia Teresa Turrini, Paulo Ricardo Bittencourt Guimarães, Maria de Fátima Mantovani, Luciana de Alcantara Nogueira, Luciana Puchalski Kalinke

**Affiliations:** 1 Universidade Federal do Paraná, Programa de Pós-Graduação em Enfermagem, Curitiba, PR, Brasil.; 2 Universidade de São Paulo, Escola de Enfermagem, Departamento de Enfermagem Médico-Cirúrgica, São Paulo, SP, Brasil.; 3 Universidade Federal do Paraná, Departamento de Estatística, Curitiba, PR, Brasil.

**Keywords:** Esencias Florales, Esperanza, Calidad de Vida, Oncología Integrativa, Cuidados Paliativos

## Abstract

**Objetivo:**

Evaluar el efecto de las Flores de Bach en la esperanza y la calidad de vida de las personas con cáncer avanzado.

**Método:**

Ensayo clínico aleatorizado, triple ciego (1:1) en un hospital público de alta complejidad en la región sur de Brasil. Noventa y nueve participantes fueron asignados al grupo de intervención (solución con nueve esencias florales) o al grupo placebo (solución de hidrobrandy). Ambos participantes utilizaron cuatro gotas por vía oral, cuatro veces al día, durante 120 días, con un seguimiento mensual. La esperanza medida por el *Herth Hope Index*, calidad de vida a través de *Functional Assessment of Chronic Illness Therapy* – *Palliative Care*.

**Resultados:**

En el grupo floral (n = 50), la esperanza varió de 43,44/48 a 44,53/48 y del grupo placebo (n+49) de 41,67/48 a 44,29/48, un aumento significativo con el tiempo (p = 0,024), sin diferencia entre los grupos (p = 0,977). La calidad de vida varió de 141,39/184 a 150,60/184 (grupo de terapia floral) y de 133,76/184 a 148,96/184 (grupo placebo), sin diferencias significativas entre los grupos (p = 0,230) ni a lo largo del tiempo (p = 0,240).

**Conclusión:**

Aunque no fue estadísticamente significativo, se observó un ligero aumento en la esperanza de vida y la calidad de vida, lo que subraya la importancia de las terapias no farmacológicas en la práctica de cuidados paliativos basados en la evidencia.

**Registro Brasileño de Ensayos Clínicos:**

RBR-8q6z6kq.

## INTRODUCCIÓN

Entre la visión dicotómica restringida de lo curable y lo incurable, la enfermedad avanzada está intrínsecamente asociada al sufrimiento y al enfrentamiento. En las etapas avanzadas del cáncer, el tratamiento paliativo (TP), los cuidados paliativos (CP) y el apoyo buscan mejorar la calidad de vida relacionada con la salud (CVRS) y prolongar la supervivencia^(1,2)^.

Independientemente del estadio de la enfermedad o de los objetivos terapéuticos, los CP tempranos están indicados por tener un enfoque centrado en la persona y sus familiares, con una perspectiva transformadora que va más allá del modelo centrado en la enfermedad y por atender las dimensiones biopsicosocioespirituales para mantener una supervivencia con calidad^(1,2,3)^.

Muchas personas reciben TP sin una integración temprana con enfoques de CP, por mucho que compartan objetivos para beneficiar a los pacientes en condiciones clínicas elegibles.

En el cáncer avanzado o incurable, se indican diferentes modalidades de TP, ya sean sistémicas (quimioterapia, hormonoterapia, inmunoterapia y terapia dirigida) o localizadas (radioterapia y cirugía), dirigidas al control de la progresión de las metástasis. Son terapias complejas, manejadas en unidades ambulatorias o hospitalizaciones por equipos transdisciplinarios, que deben ofrecerse de manera oportuna y concomitante con un enfoque de cuidados paliativos^(2)^.

En el contexto paliativo, la producción científica se orienta ampliamente a investigar y optimizar la calidad de vida relacionada con la enfermedad (CVRS), entendida como la percepción de la persona sobre el impacto de la enfermedad y el tratamiento en las dimensiones física, funcional, social, psicoemocional, existencial y de finitud^(1,3,4)^. Sin embargo, en las personas con cáncer avanzado, la esperanza sigue siendo poco investigada, destacándose en solo el 4% de las publicaciones^(3)^. La esperanza coexiste con la salud-enfermedad-cuidado crónico; es un mecanismo de afrontamiento que debe evaluarse y promoverse^(5)^.

La esperanza es la fuerza motriz para la supervivencia, no limitada por rasgos, conceptos lineales ni determinada externamente, entendida como “una fuerza vital y dinámica […] caracterizada por una expectativa confiada…”^(6)^, es “un atributo motivacional y cognitivo […] para iniciar y mantener la acción hacia el logro de la meta”^(7)^. La esperanza está relacionada con factores sociodemográficos, clínicos, terapéuticos, de calidad de vida relacionada con la salud (CVRS), cuestiones existenciales, apoyo social, psicología positiva, resiliencia y *coping*, entre otros^(5,8,9)^.

La psicooncología y la oncología integrativa se dedican continuamente a la búsqueda de intervenciones no invasivas y no farmacológicas para la salud humana con base científica, seguridad y eficacia, denominadas aquí Terapias no Farmacológicas (TNF), para ofrecer una atención holística^(4)^. Entre las 604 TNF diferentes clasificadas, las Esencias o Terapia Floral de Bach (TFB) fueron desarrolladas en Inglaterra entre los años 1928 y 1935 por el médico homeópata Edward Bach^(10)^. Son soluciones altamente diluidas en agua, extraídas de plantas y flores silvestres, que están destinadas al equilibrio psicoemocional, ya que actúan en niveles sutiles de armonización interna de la persona y el entorno^(11,12)^.

Dado que no está claro si existen residuos farmacológicos, no hay consenso sobre el mecanismo de acción de la TFB. Una de las hipótesis es que la solución ultradiluida contiene nanopartículas con información/energía sutil de las flores y tiene una acción psicomoduladora en el consciente y el inconsciente, para reequilibrar los estados emocionales^(11,13,14,15)^.

A pesar de las incertidumbres relacionadas con el mecanismo de acción de la TFB, esta es reconocida por la Organización Mundial de la Salud (1976), autorizada por la Política Nacional de Prácticas Integrativas y Complementarias (2018), con el respaldo del Consejo Federal de Enfermería. Se utiliza en la sociedad con la expectativa de obtener algún resultado; en consecuencia, algunos estudios destacan sus efectos en el trastorno por déficit de atención e hiperactividad^(13)^; para afecciones que afectan al cuerpo y la mente^(12)^; en la reducción del estrés, la ansiedad, la compulsión alimentaria y la frecuencia cardíaca^(16)^; y en la salud mental, el insomnio y el manejo de las emociones^(15)^.

A pesar del creciente interés por la oncología integrativa, son escasos los ensayos clínicos aleatorizados que evalúan el efecto de la Terapia Floral de Bach en el cáncer avanzado, con un enfoque en la esperanza y la calidad de vida. En este contexto, el objetivo de este estudio fue evaluar el efecto de la Terapia Floral de Bach en la esperanza y la calidad de vida de personas con cáncer avanzado.

## MÉTODO

### Diseño del Estudio

Ensayo clínico aleatorizado, triple ciego, controlado con placebo, realizado en el servicio de Oncología-Hematología de un hospital público en la región sur de Brasil. Se siguieron dos directrices para informar este ensayo: *Consolidated Standards of Reporting Trials* (CONSORT) y su extensión para *Nonpharmacologic Treatments* (NPT)^(17)^.

### Participantes y Muestra

De los participantes voluntarios, se incluyeron: adultos (≥ 18 años) con cáncer avanzado (estadio IV), clasificados en la escala *performance status del Eastern Cooperative Oncology Group* (PS-ECOG) de 0 a 3. Se excluyeron los casos de enfermedades linfoproliferativas, los pacientes exclusivamente en cuidados paliativos y aquellos sin capacidad para comunicarse verbalmente y/o por escrito.

El tamaño de la muestra se calculó con el *software* GPower (v 3.1.9.7) para un modelo longitudinal (2 grupos y 5 medidas), asumiendo f = 0,25, α = 5% y un poder de 95%. Teniendo en cuenta las pérdidas previstas, se planeó reclutar a 125 pacientes. El reclutamiento se llevó a cabo desde octubre de 2022 hasta febrero de 2024 y la evaluación de seguimiento hasta junio de 2024.

### Aspectos Éticos

El estudio fue aprobado por el comité de ética en investigación institucional con el dictamen n.º 5.204.355 y homologado en el Registro Brasileño de Ensayos Clínicos Aleatorios (ReBEC) con el dictamen n.º RBR-8q6z6kq. Todos los participantes recibieron información verbal y escrita y firmaron el consentimiento informado antes de su inclusión en la investigación.

### Intervención

Todos los frascos eran idénticos, de vidrio ámbar estéril, con capacidad para 30 ml, tapón perforado, precinto de seguridad, gotero con bulbo blanco y cánula de vidrio de 75 mm. Las soluciones preparadas podían contener la fórmula floral (GF) o la solución placebo (GP).

Todos estaban etiquetados de manera similar como “Investigación Floral” e incluían instrucciones de uso, con la única diferencia en la etiqueta numérica y secuencial.

Las soluciones se prepararon en una farmacia especializada, en frascos idénticos de 30 ml. Cada frasco contenía un volumen para 30 días de uso, con un margen adicional del 30%. El Grupo floral recibió hidro-brandy con nueve esencias florales de Bach añadidas, y el grupo placebo solo el hidro-brandy. Ambos grupos ingirieron cuatro gotas, cuatro veces al día, durante 120 días. Se enviaron mensajes a través de WhatsApp® durante las primeras ocho semanas para estimular la adherencia.

La solución de hidro-brandy estaba compuesta por 21 ml de agua mineral (marca Crystal®, densidad 1,0 y pH a 25 °C = 8,71) + 9 ml de conservante de brandy (marca Osborne®, 100% destilado de vino y 36% vol. alc.). La solución floral estaba compuesta por 2 gotas de la esencia madre floral (con 400 partes de brandy orgánico en una parte de esencia madre de Healing Herbs® procedente de Inglaterra). La fórmula (y las principales indicaciones) estaba compuesta por ***Cherry Plum*** (*Prunus cerasifera,* autocontrol emocional), ***Crab Apple*** (*Malus pumila,* aceptación y autoestima), ***Gorse*** (*Ulex europaeus,* fe y esperanza), ***Mimulus*** (*Mimulus guttatus*, aceptación), ***Mustard*** (*Sinapis arvensis* ***,*** melancolía), ***Olive*** (*Olea europaea,* agotamiento físico), ***Sweet Chestnut*** (*Castanea sativa*, afrontamiento de adversidades), ***White Chestnut*** (*Aesculus hippocastanum*, discernimiento) y ***Wild Oat*** (*Bromus ramosus,* propósito y sentido)^(11)^.

Ambos grupos continuaron recibiendo el tratamiento oncológico con intención paliativa, ya fuera sistémico o localizado, según la condición clínica de cada individuo.

## RESULTADOS

El resultado primario se evaluó mediante el Índice de Esperanza de Herth (HHI), el instrumento más resumido en revisiones sistemáticas sobre el constructo en oncología^(8,9)^. Se trata de una breve evaluación del autorrelato de esperanza compuesta por 12 ítems en una escala de Likert. La puntuación total varía de 12 a 48; es la suma del intervalo posible, y cuanto más alta es, mayor es la esperanza^(7)^. Se puede clasificar en tres niveles de esperanza según las puntuaciones: baja (12–23), media (24–35) o alta (36–48). La consistencia interna del instrumento, estimada mediante el α de Cronbach en la versión original, fue de 0,97 (excelente)^(7)^, y de 0,834 (buena) en su validación y adaptación cultural en Brasil^(18)^.

El resultado secundario evaluó la CVRS, con el instrumento de autorreportado *Functional Assessment of Chronic Illness Therapy -Palliative Care* (FACIT-Pal, versión 4). El FACIT-Pal se compone de las cuatro dimensiones de bienestar de la *Functional Assessment of Cancer Therapy* (FACT-G): físico (PWB: 7 ítems, 0-28 puntos); social/familiar (SWB: 7 ítems, 0-28 puntos), emocional (EWB: 6 ítems, 0-24 puntos), funcional (FWB: 7 ítems, 0-28 puntos) y una subescala de dominio paliativo (PalS: 19 ítems, 0-76 puntos)^(19)^. Los ítems se puntúan en una escala de Likert de 0 (nada) a 4 (mucho) puntos; las puntuaciones más altas reflejan una mejor CVRS. Las subescalas dan como resultado el Índice de Resultado del Ensayo (*Trial Outcome Index* – TOI: PWB + FWB + PalS = 0 a 132 puntos) y la puntuación total (0-184 puntos)^(19)^. La confiabilidad de la consistencia interna del α de Cronbach varía de buena a excelente en la versión original de las subescalas (FACT-G: 0,89; TOI: 0,91 y FACIT-Pal: 0,93)^(19)^ y en su traducción al portugués (FACT-G: 0,91)^(20)^.

Los eventos adversos se monitorearon en todas las visitas, clasificados por gravedad y relación causal. Los eventos graves implicaron la interrupción de la intervención, manteniendo el análisis por intención de tratar.

### Recolección de Datos

La recolección de datos se realizó en REDCap®. Al incluir a los participantes, se aplicaron los instrumentos, de manera presencial, para obtener: datos sociodemográficos y clínicos; esperanza y CVRS (línea de base, T_0_). En las evaluaciones de seguimiento, cada ± 30 días, se aplicaron los instrumentos de esperanza y CVRS (T_1_ a T_4_ – 120 días), ya sea de forma presencial, por teléfono o enviando un enlace al participante.

Se excluyó a los participantes ausentes ± 30 días después de T_0_ o de las últimas evaluaciones de seguimiento (T_1_ a T_4_), que no cumplieron con una adherencia superior al 70% del contenido del frasco según informe verbal, o en presencia de cualquier evento adverso relacionado.

### Aleatorización y Asignación

Se generó en el sitio web (https://ctrandomization.cancer.gov/tool/) una secuencia de asignación aleatoria con 150 números (1 para la intervención y 2 para el placebo), preservando el equilibrio de asignación 1:1 entre los grupos, procedimiento realizado por una investigadora no ciega (sin contacto con los participantes ni con el investigador de campo). La aleatorización se entregó a la farmacéutica (sin contacto con los participantes ni con el investigador de campo) responsable de la manipulación; estos códigos numéricos se utilizaron para identificar los frascos, determinar el contenido (TFB o placebo) y asegurar el enmascaramiento de la asignación (participantes e investigador de campo).

### Enmascaramiento

Se mantuvo el enmascaramiento triple (participantes, investigador de campo y estadístico) hasta el final del estudio. Una investigadora no cegada realizó la aleatorización y la farmacéutica se encargó de la preparación; ninguna de las dos tenía contacto con los participantes, el investigador de campo ni el estadístico. La farmacia de preparación se encontraba lejos del consultorio de Recolección de datos. El investigador de campo mantenía contacto con el equipo de dispensación y fue responsable de la recolección de datos. Una vez finalizada la recolección, el investigador de campo remitió los datos a la investigadora que realizó la aleatorización, quien, al conocer la asignación aleatoria, organizó los datos en los grupos A y B y los envió al estadístico. Tras los análisis, la investigadora no ciega aclaró cuál de los grupos era el floral o el placebo.

### Análisis de los Datos

Los datos capturados se exportaron desde REDCap® y se analizaron utilizando el *software* SPSS® 20. Para verificar la homogeneidad de los grupos, se utilizaron las pruebas de Chi-cuadrado o exacta de Fisher para las variables cualitativas y la prueba t de Student para las variables cuantitativas.

Los análisis siguieron el principio de intención de tratar, para minimizar el sesgo de selección tras la aleatorización, asociado al Modelo Lineal Mixto Generalizado (GLMM), debido a la necesidad de incorporar todas las evaluaciones disponibles de cada participante a lo largo del tiempo hasta el momento de la interrupción, según el principio de máxima verosimilitud^(21)^. Se adoptaron una matriz de covarianza no estructurada y el Criterio de Información de Akaike (AIC) para la selección del modelo. El nivel de significación se fijó en el 5%, con corrección de Bonferroni para comparaciones múltiples.

Las correlaciones entre las puntuaciones de esperanza y FACIT-Pal se verificaron mediante el coeficiente de Spearman. El nivel de significación se estableció en el 5%.

## RESULTADO

De los 125 pacientes elegibles, 99 fueron aleatorizados (GF = 50, GP = 49). Las pérdidas fueron mayores en las fases iniciales, sobre todo por falta de adherencia o fallecimiento. Al final del estudio, se analizaron 15 participantes en el GF y 17 en el GP (Figura 1). Las muertes se debieron a la evolución de la enfermedad o a complicaciones relacionadas con el tratamiento, sin relación con la intervención.

**Figura 1:** Flujograma de los participantes.

### Características de los Participantes

Los grupos resultaron homogéneos en cuanto a las variables sociodemográficas, clínicas y terapéuticas (p > 0,05) (Tabla 1).

**Tabla 1:** Características de los participantes del Grupo Floral (GF) y Grupo Placebo (GP) – Curitiba, PR, Brasil, 2025.

	Grupo Floral (n = 50)		Grupo Placebo (n = 49)		*p*
	n	(%)	n	(%)	
Mujeres	32	64,0	40	81,6	0,052^b^
Estado civil					0,770^b^
Casadas/En unión de hecho	26	52,0	24	49,0	
Solteras/Viudas/Divorciadas	24	48,0	25	51,0	
Educación					0,920^b^
Secundaria/Licenciatura	25	50,0	25	51,0	
Educación primaria	25	50,0	24	49,0	
Ocupación					0,310^b^
Jubilado/Pensionista	36	72,0	41	83,7	
Empleado/Autónomo/Ama de casa	12	24,0	6	12,2	
Desempleado	2	4,0	2	4,1	
Ingresos					0,520^b^
De 1 a 3 salarios mínimos	31	62,0	35	71,4	
De 4 a 10 salarios mínimos	14	28,0	9	18,4	
Otros (sin ingresos o > 10 salarios)	5	10,0	5	10,2	
Práctica Religiosa (si)	40	80,0	44	89,8	0,280^b^
Diagnóstico					0,070^b^
Mama	16	32,0	23	46,9	
Colorrectal	16	32,0	3	6,1	
Tráquea, bronquios y pulmón	4	8,0	3	6,1	
Ginecológico (útero y ovario)	4	8,0	8	16,3	
Gastroesofágico	4	8,0	3	6,1	
Páncreas/Vías biliares	3	6,0	3	6,1	
Próstata	1	2,0	3	6,1	
Otras localizaciones	2	4,0	3	6,1	
Metástasis					0,510^b^
Hepática	21	42,0	12	24,5	
Pulmón y pleura	21	42,0	15	30,6	
Ganglios linfáticos	20	40,0	21	42,9	
Ósea	17	34,0	22	44,9	
Peritoneo	12	24,0	14	28,6	
Otras localizaciones	22	44,0	17	34,7	
Autopercepción del estadio de enfermedad					0,120^b^
Inicial	11	22,0	4	8,2	
Avanzado	23	46	30	61,2	
Desconocido	16	32,0	15	30,6	
Tratamiento Paliativo					0,250^b^
Quimioterapia	46	92,0	43	87,8	
Inmunoterapia y hormonoterapia	7	14,0	9	18,4	
Cuidados paliativos	6	12,0	1	2,0	
Cirugía/Radioterapia	5	6,0	7	2,0	
Reevaluación y seguimiento	2	4,0	3	6,1	
Autopercepción sobre tratamiento					0,600^b^
Curativo	16	32,0	17	34,7	
Paliativo	13	26,0	16	32,7	
Desconocido	21	42,0	16	32,7	
Performance Status - ECOG					0,970^b^
0 e 1	43	86,0	42	87,7	
2 e 3	7	14,0	7	14,3	
Tratamientos en Salud Mental					0,340^b^
Antidepresivos	16	32,0	15	30,6	
Sedantes-hipnóticos	7	14,0	10	20,4	
Psicoterapia	1	2,0	2	7,1	
Estabilizadores del estado de ánimo	-	-	1	2,0	
Edad (años) (±DE)	56,0 ± 10,7		58,5 ± 13,6		0,310^a^
Hijos (±DE)	2,4 ± 1,5		2,2 ± 1,7		0,540^a^

DE: Desviación estándar;

^a^Prueba de Mann-Whitney;

^b^Prueba exacta de Fisher.

## RESULTADOS

La esperanza presentó puntuaciones elevadas desde el inicio del estudio, con una variación discreta a lo largo del mismo en ambos grupos. Según el modelo estadístico GLMM, tras aplicar la corrección de Bonferroni para comparaciones múltiples, se observó un efecto del tiempo (p = 0,024), pero no entre los grupos (p = 0,97) (Figura 2).

**Figura 2:** Evaluación del resultado en la esperanza.

En la CVRS, se observó una mejora discreta en todos los dominios en el grupo de control, pero solo el bienestar social (p = 0,022) y las preocupaciones adicionales (p = 0,049) mostraron significación a lo largo del tiempo, sin diferencias entre los grupos (Tabla 2).

**Tabla 2:** Análisis de los resultados en cuanto a calidad de vida relacionada con la salud (FACIT-Pal) – Curitiba, PR, Brasil, 2025.

Grupo	Evaluación	n	Bienestar físico (PWB)			Bienestar social (SWB)				Bienestar emocional (EWB)			Bienestar funcional (FWB)		
			Media	DE	p	Media	DE	p	Media	DE	p	Media	DE	p
Floral	T_0_ (Línea de base)	50	22,36	4,77	0,225^1^ 0,168^2^ 0,317^3^	19,86	5,13	0,160^1^ 0,022^2^ 0,174^3^	19,46	4,41	0,229^1^ 0,117^2^ 0,256^3^	18,76	6,04	0,885^1^ 0,710^2^ 0,974^3^
	T_1_ (30 días)	27	22,41	5,55		19,78	5,40		19,93	5,05		19,19	6,22	
	T_2_ (60 días)	18	22,94	5,34		22,11	4,60		21,22	2,62		19,61	5,65	
	T_3_ (90 días)	19	22,74	5,75		20,26	5,76		20,84	3,79		20,33	6,32	
	T_4_ (120 días)	15	23,07	5,51		22,00	5,92		20,53	4,29		20,60	7,32	
Placebo	T_0_ (Línea de base)	49	21,06	5,35		18,79	5,33		18,53	4,78		17,90	5,46	
	T_1_ (30 días)	33	21,52	5,52		18,59	5,11		19,00	3,78		18,88	4,17	
	T_2_ (60 días)	25	21,68	4,81		19,75	4,02		18,97	3,66		19,12	4,39	
	T_3_ (90 días)	20	19,40	5,86		19,55	4,62		19,05	3,41		19,05	4,49	
	T_4_ (120 días)	17	22,82	3,47		19,31	4,37		21,29	2,64		20,12	4,37	
			**Preocupaciones adicionales (PalS)**				**FACT-G**			**TOI**			**FACIT-Pal**		
Floral	T_0_ (Línea de base)	50	60,94	10,43	0,433^1^ 0,049^2^ 0,559^3^	80.44	16.29	0,156^1^ 0,323^2^ 0,972^3^	102,07	18,52	0,330^1^ 0,405^2^ 0,788^3^	141,39	25,50	0,230^1^ 0,240^2^ 0,877^3^
	T_1_ (30 días)	27	59,93	13,00		81.30	19.91		101,52	23,25		141,23	32,08	
	T_2_ (60 días)	18	62,89	9,96		85.89	15.83		105,44	18,81		148,78	24,70	
	T_3_ (90 días)	19	62,46	9,63		84.18	18.86		105,53	19,93		146,63	27,79	
	T_4_ (120 días)	15	64,40	11,72		86.20	21.33		108,07	23,53		150,60	32,69	
Placebo	T_0_ (Línea de base)	49	57,48	10,49		76.28	15.94		96,44	18,77		133,76	25,45	
	T_1_ (30 días)	33	59,91	8,99		77.98	13.20		100,30	15,18		137,89	20,11	
	T_2_ (60 días)	25	60,20	9,07		79.52	11.64		101,00	15,66		139,72	19,57	
	T_3_ (90 días)	20	60,75	7,85		77.05	11.95		99,20	13,27		137,80	18,01	
	T_4_ (120 días)	17	65,41	5,33		83.54	9.88		108,35	10,64		148,96	13,93	

^1-3^Valor de p basado en el modelo estadístico GLMM tras la corrección de Bonferroni para comparaciones múltiples post hoc con respecto al grupo^1^,

el tiempo^2^

y el grupo frente al tiempo^3^.

La puntuación de esperanza y la CVRS se correlacionaron positivamente (p < 0,05) con todos los dominios, subescalas y puntuación total en ambos grupos. Estos constructos están fuertemente relacionados, con niveles elevados de esperanza asociados a los diferentes dominios de la CVRS (Tabla 3).

**Tabla 3:** Correlación entre la puntuación de esperanza (HHI) y la CRVS (FACIT-Pal) – Curitiba, PR, Brasil, 2025.

Dominios, subescalas y puntuación total	Grupo Floral (n = 50)	p	Grupo Placebo (n = 49)	p
	Spearman R		Spearman R	
Bienestar físico (PWB)	0,427	0,002	0,367	0,010
Bienestar social (SWB)	0,415	0,003	0,503	0,000
Bienestar emocional (EWB)	0,653	0,000	0,496	0,000
Bienestar funcional (FWB)	0,465	0,001	0,473	0,001
FACT-G	0,606	0,000	0,572	0,000
Preocupaciones adicionales (PalS)	0,503	0,000	0,694	0,000
TOI	0,512	0,000	0,645	0,000
FACIT-Pal	0,560	0,000	0,671	0,000

Abreviaturas: FACT-G, Evaluación Funcional de la Terapia Oncológica - General; TOI, Índice de Resultados de Ensayos Clínicos; FACIT-Pal, Evaluación Funcional de la Terapia para Enfermedades Crónicas - Cuidados Paliativos.

## DISCUSIÓN

En este ensayo clínico aleatorizado, triple ciego y controlado con placebo, la TFB no mostró un efecto superior al placebo sobre la esperanza de vida y la calidad de vida relacionada con la salud (CVRS) en personas con cáncer avanzado en fase terminal.

La ausencia de diferencias significativas entre los grupos puede explicarse por múltiples factores. En el contexto de la investigación en cuidados paliativos, se reconoce que el seguimiento sistemático, las evaluaciones periódicas y el apoyo ofrecido por el equipo de investigación funcionan como intervenciones en sí mismas, produciendo un efecto placebo que refuerza los sentimientos de esperanza y mejora la percepción de la calidad de vida^(3,14)^.

La síntesis de un metaanálisis con múltiples tipos y estadios de cáncer durante la terapia sistémica identificó que 35,6 es la media de la puntuación de esperanza^(8)^. Nuestros hallazgos apuntan a una superioridad de aproximadamente 9 puntos en personas con cáncer avanzado en TP. Se trata de una condición desafiante en la que estas personas desarrollan estrategias de afrontamiento, visualización, planificación y ejecución de acciones para alcanzar resultados más favorables, que son esenciales en la lucha contra la desesperanza y la promoción de pensamientos positivos, incluso ante la progresión de la enfermedad avanzada y las limitaciones clínicas^(22)^.

El punto de corte establece que >40 se considera una puntuación elevada de esperanza en personas con cáncer y que la relevancia clínica de una intervención tendría una diferencia superior a cinco puntos^(5)^. Cabe destacar que, en nuestros hallazgos, los participantes ya presentaban puntuaciones elevadas en la línea de base, lo que limita la posibilidad de ganancias adicionales y caracteriza un posible efecto techo en la puntuación de esperanza^(5)^. La esperanza es un constructo complejo, influenciado por dimensiones sociodemográficas, clínicas, existenciales y relacionales, lo que dificulta la detección de cambios significativos a partir de una sola intervención; la mayoría de los estudios con efectos moderados a pequeños se basaron en la teoría de la esperanza o en la planificación anticipada de cuidados^(5,23)^.

La escasez de estudios en pacientes con cáncer realizados con la TFB limita las comparaciones. El único estudio experimental disponible utilizó una fórmula (*Gorse, Star of Bethlehem, Gentian, Mimulus, Wild Oat, Honeysuckle y Heather*) y se llevó a cabo en pacientes cubanos; la significación estadística en el GF en comparación con el tratamiento convencional con antidepresivos se pone en duda debido al alto riesgo de sesgo^(24)^.

Además de la elevada puntuación de esperanza, la presente investigación identificó percepciones imprecisas sobre el diagnóstico y los objetivos del tratamiento. Las personas con cáncer avanzado sucumben a diversos sesgos cognitivos y son más propensas a creer que la enfermedad es curable y que tendrán una mayor supervivencia^(23)^. Por lo tanto, cabe preguntarse si la imprecisión del conocimiento diagnóstico y terapéutico pudo haber influido en la sobrevaloración de la esperanza y de la CVRS.

Mantener o mejorar la CVRS y la supervivencia son los objetivos de las diferentes modalidades de TP. Las personas con cáncer avanzado se adaptan constantemente a la cronicidad de la enfermedad, experimentan particularidades distintas de la fase inicial o de la terminalidad, y atraviesan una transformación conductual, psicológica y existencial^(2,25)^. Se trata de una fase impregnada de procesos internos que resultan en sufrimiento físico y psicoemocional^(9)^. Con frecuencia, antes de la TP estos pacientes ya presentaban un deterioro en diferentes dominios de la CVRS, principalmente en los físicos, emocionales y sociales, los cuales no siempre se optimizarán después del tratamiento^(26)^.

El resultado de este estudio clínico difiere de la literatura internacional, al encontrar puntuaciones superiores en diferentes dominios, subescalas y en el total del FACIT-Pal^(27)^. La presencia de puntuaciones elevadas de CVRS en la línea de base puede reflejar el efecto techo, en el que los participantes con mejores puntuaciones en el dominio funcional reducen la sensibilidad para detectar pequeñas variaciones en el seguimiento y estas no se captan como estadísticamente significativas. Aunque se desconocen los umbrales de cambio clínicamente significativo para el FACIT-Pal, se ha sugerido una diferencia media inferior a 5 puntos, dada la complejidad para detectar diferencias robustas en esta población^(28)^.

A pesar de la escasez de estudios en personas con cáncer, la intervención presenta resultados positivos en la CVRS en otras poblaciones, como en el estudio cuasi-experimental que evaluó el efecto de la TFB (*Rescue Remedy*® y*Walnut)* en profesionales de enfermería en Brasil, con resultados significativos en la subescala de estrés traumático después de tres semanas^(29)^. Otra fórmula (*Rescue Remedy*®, *Red Chestnut, Aspen, Crab Apple, Oak y Olive*), utilizada en el estudio de métodos mixtos en profesionales de la salud españoles, muestra resultados significativos en las dimensiones de salud mental, insomnio y control emocional^(15)^.

En el proceso terapéutico con TFB, las personas posiblemente acceden a patrones emocionales y mentales profundos de la conciencia, los cuales pueden causar malestar o negación, lo que hace que acepten o rechacen las transformaciones emocionales^(11,30)^. En algunas situaciones, las personas pueden presentar un empeoramiento de los signos y síntomas, hecho que posiblemente se explique por la catarsis, en la que se produce inicialmente la liberación de emociones negativas (tristeza, ira, miedo o frustración), para una posterior mejora del bienestar emocional^(11)^. Este hecho puede percibirse en la oscilación de las rectas de regresión de la puntuación de esperanza, en los dominios de bienestar social y preocupaciones adicionales. Es importante considerar que el efecto terapéutico no es uniforme ni automático, lo que puede dar lugar a la ausencia de significación debido a una menor diferencia entre el GF y el GP^(30)^.

En esta investigación, se identificó un elevado número de pérdidas de seguimiento de participantes (adhesión insuficiente o no percepción del efecto). En otro estudio clínico pragmático, en Brasil, no se identificó entre los participantes la expectativa de “ninguna mejoría” sobre la TFB. Este hecho puede influir en la percepción de resultados positivos en ambos grupos y, asociado a la baja adherencia de los participantes durante el estudio, refleja la realidad de esta intervención^(14)^, aunque una revisión sistemática de métodos mixtos identifica diferentes barreras en cuanto al uso de las TNF, desde el desconocimiento por parte de la población, la falta de financiamiento, la escasa evidencia y la baja receptividad de los profesionales de la salud^(10)^.

El uso de la TFB en estudios clínicos^(14,15,16,24,30)^, revisiones sistemáticas^(13)^ y estudios exploratorios^(12)^ difiere de lo observado en este estudio en cuanto a la ausencia de eventos adversos. Cabe destacar que en la composición de la fórmula se utilizó un conservante con bajo contenido de alcohol. Sin embargo, existen limitaciones para establecer una relación causal, ya que los efectos pueden estar relacionados con las características clínicas de esta población, múltiples toxicidades previas, uso de quimioterapéuticos, comorbilidades y uso de polifarmacia.

Este estudio presenta limitaciones, entre ellas una muestra predominantemente femenina con cáncer de mama, lo que restringe la generalización de los hallazgos. Otros tipos de cáncer y los pacientes del sexo masculino pueden experimentar la esperanza y la CVRS de manera diferente. Además, la inclusión de participantes con comorbilidades psiquiátricas, sin análisis por subgrupos, puede haber influido en los resultados. Otro punto relevante se refiere a la fórmula floral. De acuerdo con las recomendaciones de Bach, las fórmulas deben personalizarse para satisfacer las necesidades y especificidades de cada individuo. Bach creía que las flores promovían estados emocionales positivos al catalizar los recursos internos del paciente, contribuyendo a la restauración del equilibrio emocional^(11)^.

Otra limitación es que no fue posible garantizar un control riguroso del cumplimiento de la dosis recomendada. El uso durante 120 días pudo haber sido excesivo y, asociado al retraso en la percepción sutil del efecto de la TFB, pudo haber contribuido al bajo cumplimiento. El uso de brandy (conservante alcohólico), incluso en baja concentración, pudo haber sido responsable de los eventos adversos leves reportados.

## CONCLUSIÓN

La Terapia Floral de Bach, en la formulación evaluada (*Cherry Plum, Crab Apple, Gorse, Mimulus, Mustard, Olive, Sweet Chestnut, White Chestnut y Wild Oat*), no presentó un efecto superior al placebo sobre la esperanza de vida o la calidad de vida relacionada con la salud en pacientes con cáncer avanzado. Aunque ambos grupos mostraron un discreto aumento en las puntuaciones evaluadas, este resultado puede reflejar el efecto placebo y la atención clínica recibida. Junto con los eventos adversos leves notificados, los hallazgos también sugieren precaución en el uso de conservantes alcohólicos en esta población.

Estos hallazgos refuerzan la necesidad de ensayos clínicos sólidos, con intervenciones personalizadas, un mejor seguimiento de la adherencia y el uso de medidas psicométricas sensibles. Para la enfermería, el estudio contribuye a fortalecer la práctica basada en la evidencia en los cuidados paliativos y a ampliar la reflexión sobre el papel de las terapias no farmacológicas.

## DISPONIBILIDAD DE DATOS

Todo el conjunto de datos que apoya los resultados de este estudio fue publicado en el propio artículo.
